# Co-immobilization of Ciprofloxacin and Chlorhexidine as a Broad-Spectrum Antimicrobial Dual-Drug Coating for Poly(vinyl chloride) (PVC)-Based Endotracheal Tubes

**DOI:** 10.1021/acsami.4c01334

**Published:** 2024-03-20

**Authors:** Diana Filipa Alves, Maria Olívia Pereira, Susana Patrícia Lopes

**Affiliations:** † CEB - Centre of Biological Engineering, University of Minho, 4710-057 Braga, Portugal; ‡ LABBELS—Associate Laboratory, 4710-057 Braga/Guimarães, Portugal

**Keywords:** co-immobilization, endotracheal tube, dual-drug coating, polymicrobial biofilm, ventilator-associated pneumonia

## Abstract

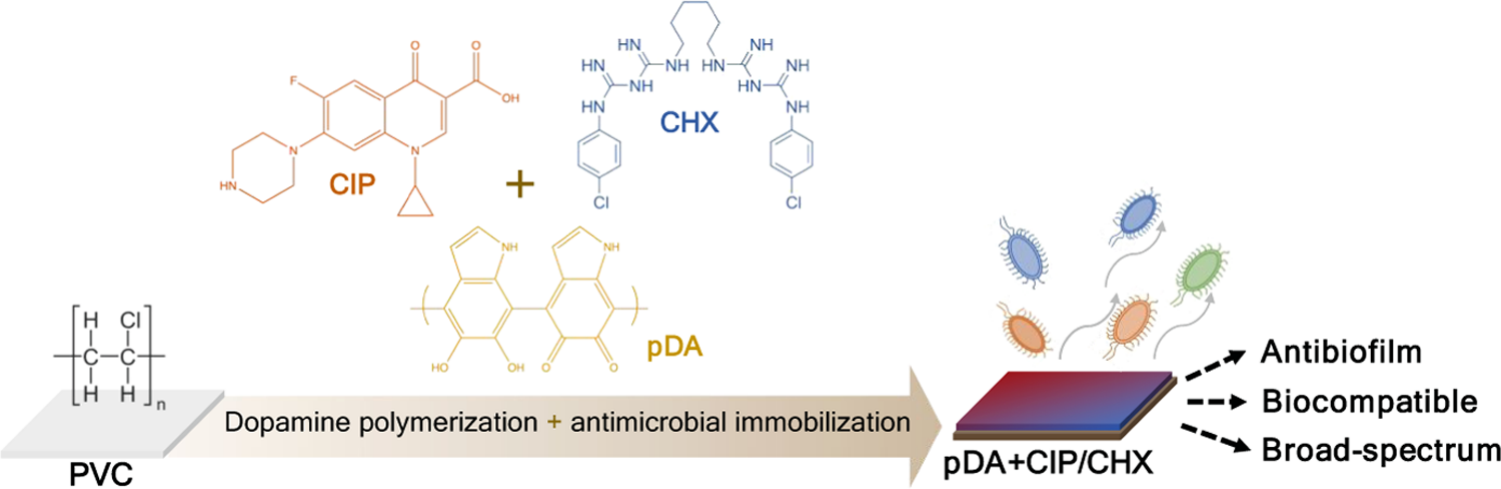

The endotracheal tube (ETT) affords support for intubated patients, but the increasing incidence of ventilator-associated pneumonia (VAP) is jeopardizing its application. ETT surfaces promote (poly)microbial colonization and biofilm formation, with a heavy burden for VAP. Devising safe, broad-spectrum antimicrobial materials to tackle the ETT bioburden is needful. Herein, we immobilized ciprofloxacin (CIP) and/or chlorhexidine (CHX), through polydopamine (pDA)-based functionalization, onto poly(vinyl chloride) (PVC) surfaces. These surfaces were characterized regarding physicochemical properties and challenged with single and polymicrobial cultures of VAP-relevant bacteria (*Pseudomonas aeruginosa*, *Acinetobacter baumannii*, *Klebsiella pneumoniae*, *Staphylococcus aureus*, *Staphylococcus epidermidis*) and fungi (*Candida albicans*). The coatings imparted PVC surfaces with a homogeneous morphology, varied wettability, and low roughness. The antimicrobial immobilization via pDA chemistry was still evidenced by infrared spectroscopy. Coated surfaces exhibited sustained CIP/CHX release, retaining prolonged (10 days) activity. CIP/CHX-coated surfaces evidencing no A549 lung cell toxicity displayed better antibiofilm outcomes than CIP or CHX coatings, preventing bacterial attachment by 4.1–7.2 Log_10_ CFU/mL and modestly distressing*C. albicans*. Their antibiofilm effectiveness was endured toward polymicrobial consortia, substantially inhibiting the adhesion of the bacterial populations (up to 8 Log_10_ CFU/mL) within the consortia in dual- and even in*P. aeruginosa*/*S. aureus*/*C. albicans* triple-species biofilms while affecting fungal adhesion by 2.7 Log_10_ CFU/mL (dual consortia) and 1 Log_10_ CFU/mL (triple consortia). The potential of the dual-drug coating strategy in preventing triple-species adhesion and impairing bacterial viability was still strengthened by live/dead microscopy. The pDA-assisted CIP/CHX co-immobilization holds a safe and robust broad-spectrum antimicrobial coating strategy for PVC-ETTs, with the promise laying in reducing VAP incidence.

## 1 Introduction

The success of current clinical practice is highly dependent on the use of medical devices or implants. In this regard, the endotracheal tube (ETT) has been an elemental device in providing airway patency in patients requiring mechanical ventilation, e.g., in cases like surgeries using general anesthesia, critical care situations, or a traumatically compromised airway.^1^ Mechanical ventilation has long been acknowledged as a life-support practice that has picked up during the latest COVID-19 pandemic, given the unprecedented number of patients that had to be admitted to intensive care units (ICUs) with severe symptoms of respiratory failure.^2^ Once placed, the ETT becomes potentially harmful to critically ill patients undergoing artificial ventilation, as it impairs natural defense mechanisms (e.g., cough reflex, upward mucosal ciliated transport, clearance mechanism operative in the airway) and causes mechanical tissue irritation due to breathing cycles.^3^ Furthermore, the ordinary ETTs, which are often made of flexible materials [e.g., poly(vinyl chloride), PVC], are devoid of antimicrobial activity, being continuously exposed to ambient air and providing an ideal source for microbial colonization and the development of a so-called biofilm, both early and frequent phenomena contributing to the development of ventilator-associated pneumonia (VAP). Indeed, microorganisms reach the ETT’s distal end due to contaminated oropharyngeal contents or, eventually, gastric secretion reflux.^4^ Accumulation and coaggregation of common commensal bacteria further promote the recruitment of opportunistic nosocomial pathogens, developing jointly a biofilm with serious implications, ultimately leading to VAP.^5^ The ETT biofilm correlates with VAP pathogenesis, as microbial cells encase themselves in a self-produced matrix of extracellular polymeric substances, conferring them protection against antimicrobial treatments and the host immune system.^6^ The diversity of organisms readily colonizing the ETT surface, therefore, involves not only drug-resistant top-priority pathogens from the ESKAPE bacterial panel such as*Pseudomonas aeruginosa*, *Acinetobacter baumannii*, *Klebsiella pneumoniae*, or *Staphylococcus aureus* but also others that may include commensal bacteria (*Staphylococcus epidermidis*) or even fungi (*Candida albicans*) with pathogenic potential, all contributing to the polymicrobial and recalcitrant nature of ETT biofilms.^7,8^

VAP is the commonest life-threatening nosocomial infection in the developed world, accounting for an estimated 22% of prevalence^9^ and contributing to up to 50% of all cases in ICUs.^10^ Its discrepant morbidity/mortality rates (16–44%), ICU/hospital length of stay (13–26.6 days), and incidence range (11–84%), with ICU antibiotic prescriptions near 50%, are all alarming indicators of clear action needed.^11^ Given the difficulties associated with finding an effective treatment for VAP, reducing ETT bioburden through ETT surface′ modification is likely the most suitable approach to avoid VAP. Despite years of technological progress, there are still limited reports on ETT coatings. Overall, studies have employed active (directly compromising surface microbial colonization), passive (contamination-resistant surface with altered composition and/or pattern), or combinatorial antimicrobial and antifouling approaches but most limitedly addressing antimicrobial activity and/or safety of use.^11^ In addition, studies have proven insufficient in tackling polymicrobial ETT biofilms, an underappreciated issue with a significant fate for VAP. Therefore, the development of pertinent, that is, suitable and highly efficient materials endowed with a broad-spectrum antimicrobial/antibiofilm activity, biocompatibility, and cost-effectiveness features, tends to be a gold solution for the next generation of ETTs, with promise in preventing VAP.

Within the wide range of surface functionalization strategies, the bioinspired polydopamine (pDA) coating approach stands out as one that fulfills all of the aforementioned requirements, tailoring a vast variety of materials^12−14^ serving a broad range of applications, including biomedical. The great interest in pDA-based coatings is due to their ease of preparation, wherein substrata are simply immersed in an alkaline solution of dopamine that then self-polymerizes and gives rise to an adhesive film with a thickness typically in the nanometer range. Alongside its simple processing conditions, other unique features such as material independence, strong reactivity for secondary functionalization, and high biocompatibility render pDA extremely interesting for immobilization of various antimicrobials, endowing biomaterials’ surfaces with anti-infective properties.^14,15^ Chlorhexidine (CHX) has been extensively applied in different hospital protocols for oral hygiene of intubated patients to reduce the incidence of VAP.^16^ CHX immobilization has shown great potential while imparting metallic surfaces with antimicrobial traits against Gram-positive bacteria in the context of orthopedic infections.^14^ Impregnation of ETTs with antiseptics, including CHX, has also led to auspicious antimicrobial outcomes toward drug-resistant bacteria and fungi.^17,18^ In turn, ciprofloxacin (CIP) is a second-generation quinolone with a proven broad-spectrum activity against both Gram-negative and Gram-positive bacteria. Guidelines have recommended CIP as a second antipseudomonal agent in dual-combination regimens in VAP management,^19^ thus holding promise to be further immobilized on ETT surfaces. Furthermore, mono- and multidrug-loaded coatings (not pDA-assisted) have been developed in varied contexts. CIP has been combined with azithromycin to impregnate urinary catheters yielding promising results *in vitro* against *P. aeruginosa* ,^20^ and it also demonstrated long-term *in vivo* effectiveness in a *P. aeruginosa* urine model.^21^ Another approach comprises the incorporation of CIP together with other antibiotics on biodegradable polymers that were then applied on metallic implants to act as a drug delivery system, locally releasing the antibiotics.^22^

In this study, the pDA-assisted co-immobilization of CIP and CHX was made on PVC surfaces to devise a novel and safe ETT coating supporting antimicrobial activity against a wide range of VAP-relevant species that include bacteria and fungi. PVC surfaces were physically and chemically characterized (morphology, roughness, wettability, molecular composition) and inspected for their effectiveness in inhibiting the development of single-, dual-, and triple-species biofilms without compromising the viability of A549 lung epithelial cells.

## 2 Materials and Methods

### 2.1 Microbial Strains and Culture Conditions

Five model reference bacterial strains and one fungal strain were used throughout this study, namely, *P. aeruginosa* ATCC 27853, *K. pneumoniae* ATCC 11296, *S. aureus* ATCC 25923 (all from American Type Culture Collection), *A. baumannii* NIPH 501^T^ (kindly provided by Professor Alexandr Nemec, National Institute of Public Health in Prague), and *S. epidermidis* CECT 4183 (from the Spanish Type Culture Collection). The fungal reference strain *C. albicans* SC5314 was also used. All bacterial/fungal strains were stored at 80 ± 2 °C in a broth medium with 20% (v/v) glycerol.

All microbial strains were plated from frozen stock solutions and first streaked plates containing tryptic soy agar (TSA, Liofilchem) or Sabouraud dextrose agar (SDA, Liofilchem), respectively, for bacteria and fungi. After incubation (37 °C for ± 24 h), some colonies were collected from the agar plates and grown overnight in batches of tryptic soy broth or Sabouraud dextrose broth (TSB/SDB, Liofilchem) at 37 °C under agitation (120 rotations per minute, rpm) on a horizontal shaker (Biosan OS-20). Bacterial and fungal cells were harvested by centrifugation (9000*g*, 5 min) and washed in sterile saline solution (0.9% w/v NaCl) or phosphate-buffered saline (PBS, pH 7.4), respectively. The concentration of bacterial suspensions was then adjusted by measuring the absorbance at 620 nm (EZ Read 800 Plus, Biochrom) and using previously established standard curves, while the fungal suspension concentration was adjusted after the cell numbers were estimated in a Neubauer counting chamber.

### 2.2 Preparation of Antimicrobial Stock Solutions

Stock solutions of the antimicrobial agents, CIP and CHX (both from Sigma-Aldrich, St. Louis, MO), were prepared by dissolving the powders in hydrochloric acid (HCl 0.2 M) or absolute ethanol and further diluted in growth medium or PBS, depending on the procedure performed (susceptibility testing and compound release, respectively). Storage was according to the manufacturer’s instructions.

### 2.3 PVC Preparation and Functionalization

**Scheme 1:**
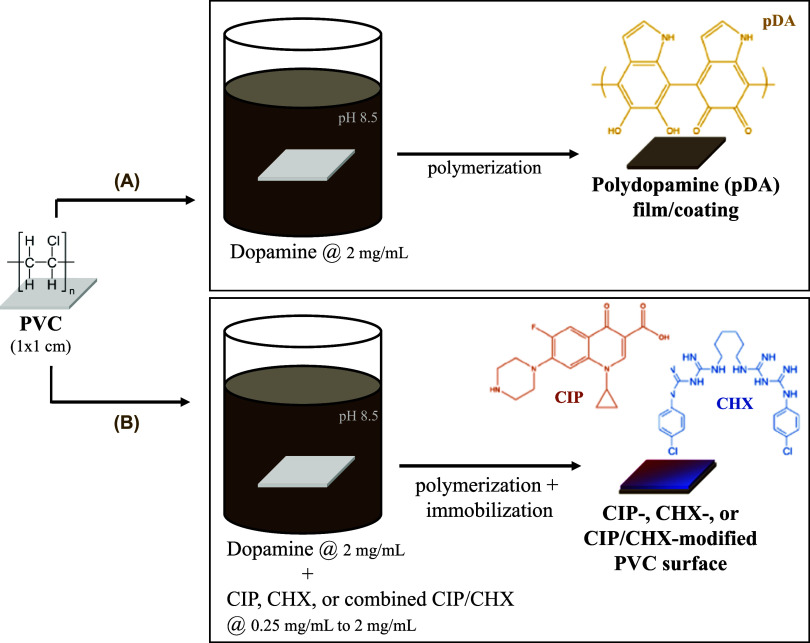
Scheme of the Methodology Used in the PVC Functionalization with CIP, CHX and CHX-CIP (A) PVC immersion in an alkaline solution (pH 8.5) of dopamine (at 2 mg/mL) resulted in its polymerization and subsequent deposition of an adhesive film called polydopamine (pDA). (B) Antimicrobial immobilization was performed by immersion of PVC on dopamine (at 2 mg/mL) combined with each or both compounds (at 0.25 mg/L to 2 mg/mL), resulting in CIP-, CHX-, or CIP/CHX-modified PVC surfaces upon dopamine polymerization and antimicrobial(s) immobilization. All pDA, CIP, and CHX chemical structures were designed using the ChemDraw Professional for Mac (version 16.0.1.4, CambridgeSoft).

For surface functionalization, 1 mm thickness unplasticized sheets of PVC (purchased from Goodfellow Cambridge Ltd., Cambridgeshire, U.K.) were cut into 1 × 1 cm squared coupons. Before surface modification, coupons were subjected to an ultrasonic cleaning treatment in a commercial detergent (Sonasol, Henkel Ibérica, Portugal) for 5 min to remove any traces of impurities and grease. After being thoroughly rinsed with distilled water, cleaned PVC surfaces were sterilized with 70% v/v ethanol for 30 min, rinsed with sterile ultrapure water, and finally irradiated with ultraviolet (UV) light for 1 h. To impart PVC surfaces with antimicrobial features, PVC functionalization with CIP and/or CHX was performed using a previously reported one-step pDA-assisted approach for the immobilization of CHX on stainless steel,^14^ the so-called mussel-inspired coating strategy. The methodology scheme used for PVC functionalization is depicted in Scheme 1. Briefly, PVC coupons were first immersed in 7 mL of a 2 mg/mL solution of dopamine (Sigma-Aldrich) freshly prepared in 10 mM bicine buffer pH 8.5 (Sigma-Aldrich), for 18 h at room temperature under agitation (70 rpm). After dopamine polymerization, a pDA film was formed on the PVC surface (Scheme 1A). Regarding PVC functionalization with the antimicrobials, 2 mg/mL of dopamine was added to CHX and/or CIP (that is, alone or combined) at concentrations ranging from 0.25 to 2 mg/mL, which were dissolved together in 10 mM bicine buffer, pH 8.5. The PVC coupons were simply immersed in these solutions, following the above-mentioned conditions. PVC surfaces modified with CIP, CHX, or CIP/CHX combined were obtained following dopamine polymerization and antimicrobial immobilization (Scheme 1B). All modified PVC surfaces were then rinsed by immersion in a Petri dish filled with sterile ultrapure water, for the same duration, ensuring consistency in the resulting surfaces, and air-dried for further application. With this functionalization technique, it was expected that compounds would be incorporated throughout the full thickness of the pDA film.^23^ For comparison purposes, unmodified PVC (before pDA coating or antimicrobial immobilization) and pDA-coated surfaces were used in further assays.

### 2.4 Antimicrobial Activity of Immobilized Compounds

To inspect whether CHX and CIP retained antimicrobial activity upon (co-)immobilization, the antimicrobial properties of the modified surfaces were performed as previously reported.^14^ Briefly, each microbial suspension was adjusted to ∼1 × 10^6^ CFU/mL as described above, and 20 μL of the microbial culture was placed at the top of each unmodified/modified PVC surface and incubated at 37 °C (static conditions). The suspension was allowed to dry on the top of unmodified/modified PVC surfaces and then placed with the face exposed to microbial culture in contact with agar (TSA/SDA). Agar plates were then incubated at 37 °C for 24 h, and the presence or absence of microbial growth on the agar was further inspected through visual observation. The contact antimicrobial activity of CIP- and CHX-modified PVC surfaces was also evaluated using an adaptation of the Kirby–Bauer disc diffusion method.^24^ For that, modified (vs unmodified) surfaces were placed on top of TSA/SDA plates previously streaked with each microbial suspension adjusted to ∼1 × 10^8^ CFU/mL, which were then incubated (37 °C) for up to 72 h. The presence or absence of an inhibition zone was further investigated and annotated.

### 2.5 Surface Characterization

#### 2.5.1 Surface Morphology

Surface morphology was observed by scanning electron microscopy (SEM) using an ultrahigh-resolution field emission gun SEM (FEG-SEM, NOVA 200 Nano SEM, FEI Company). Before analysis, samples were first covered with a very thin film (5 nm) of Au–Pd (80–20 wt %). Topographic images were obtained with a secondary electron detector using the following parameters: an acceleration voltage of 10 kV, a ∼5 mm stage distance, and 10,000 and 50,000 × magnifications.

#### 2.5.2 Surface Roughness

Atomic force microscopy (AFM) measurements were performed at room temperature using a CSI—Nano-Observer atomic force microscope, operating in tapping mode. The scanning area per sample was fixed at 10 μm × 10 μm at a 512-pixel resolution. Surface morphology and roughness analyses were then conducted using Gwyddion software.

#### 2.5.3 Surface Wettability

Surface wettability was investigated by measuring the static water contact angle using a sessile drop method in an automated contact angle measurement apparatus (OCA 15 Plus, Dataphysics, Germany) that allows image acquisition and data analysis. Contact angles were measured using 3 μL drops of ultrapure water.

#### 2.5.4 Surface Molecular Composition

The molecular composition of the surfaces was analyzed by infrared spectroscopy. Fourier transform infrared (FTIR) spectra of the surfaces were acquired with an FTIR SpectrumTwo (PerkinElmer) in attenuated total reflectance mode (ATR) with a platinum accessory in the wavenumber range of 4000–500 cm^–1^, using 64 scans at a resolution of 4 cm^–1^.

#### 2.5.5 CHX and CIP Release Profiles

To ascertain the release profiles of both antimicrobial compounds (CIP and CHX), the CIP- and CHX-modified PVC coupons were placed in 6-well microtiter plates (Orange Scientific) to which 4 mL of PBS (pH 7.4) was added and then incubated at 37 °C under constant agitation at 120 rpm. The PBS was all withdrawn and refreshed at different time points (every 24 h for up to 10 days). The amount of released CHX or CIP was then determined by UV–visible spectroscopy (UV–vis), measuring the absorbance at 255 and 270 nm, respectively. Data was then converted to concentration values by using previously established calibration curves. The remaining contact antimicrobial activity after 4 and 10 days of release was determined as described in Section 2.4.

### 2.6 Cytotoxicity Determination

Cytotoxicity was evaluated on human lung epithelial A549 cells (ATCCCCL-185, according to the ISO 10993-5:2009.^25^ Cells were grown in Dulbecco’s modified Eagle’s medium (DMEM, Biochrom) supplemented with 10% fetal bovine serum (FBS, Gibco) and 1% protective antibiotic solution (ZellShield, Biochrom) at 37 °C and 5% CO_2_. Once confluence was achieved, cells were detached using trypsin-ethylenediaminetetraacetic acid (EDTA) solution (0.25/0.02%) in PBS without Ca^2+^ and Mg^2+^ (PAN Biotech GmbH, Germany) and 100 μL of cell suspension adjusted to 1 × 10^5^ cells/mL was transferred per well to a 96-well microtiter plate. In parallel, unmodified/modified PVC surfaces were inserted in 24-well plates, and 1 mL of supplemented DMEM was added to each well. Both plates with cells and surfaces were incubated at 37 °C and 5% CO_2_ for 24 h. After this period, the supernatant was removed, and 100 μL of the medium that was in contact with the surfaces was added. Fresh-supplemented DMEM was also added as a positive control. The plate was then incubated (37 °C; 5% CO_2_) for an additional 24 h. In the dark, 20 μL of 3-(4,5-dimethylthiazol-2-yl)-5-(3-carboxymethoxyphenyl)-2-(4-sulfophenyl)-2*H*-tetrazolium (MTS) inner salt (Promega) was added to each well, and the plate was further incubated for 1 h at 37 °C, 5% CO_2_. The absorbance of the resulting solution was measured at 490 nm. The percentage of cell viability was calculated by the absorbance ratio between the cell growth in the presence of the coating and the control growth (cell growth in supplemented DMEM).

### 2.7 Antibiofilm Performance of Modified PVC Surfaces against Single-Species and Polymicrobial Consortia

The ability of the prepared modified surfaces to prevent biofilm formation was evaluated by CFU counting against single species and polymicrobial cultures encompassing the aforementioned bacterial and fungal species. Overnight cultures were used to prepare microbial inocula with approximately 1 × 10^6^ CFU/mL in TSB (bacterial cultures) or RPMI 1640 (fungal culture). For dual- and triple-species consortia, a proportion of 1:1 and 1:1:1, respectively, of each suspended bacterial/fungal inoculum was used. Unmodified- and modified PVC-squared coupons were then inserted into a 24-well microtiter plate (Orange Scientific) and inoculated with 1 mL of microbial suspension prepared as described. As stated above, unmodified PVC and pDA-coated surfaces were used for comparison purposes. The plates containing the coupons were kept at 37 °C for 24 h under static conditions. Coupons were then washed twice with a saline solution (for bacteria) or PBS (for fungi) and transferred to new wells filled with 1 mL of saline solution or PBS for further biofilm cell quantification. Adhered bacterial cells were removed from the PVC coupons by ultrasonic bath treatment in a Sonicor SC-52 (Sonicor Instruments) operating at 50 kHz for 6 min, while fungal-adhered cells were removed by scrapping the surfaces (parameters previously optimized). The biofilm samples, after detached from the surfaces, were afterward collected, vortexed to disrupt possible cell aggregates, 10-fold serially diluted, and plated onto appropriate agar plates that were incubated for 16–48 h at 37 °C in an aerobic incubator before biofilm cell enumeration. Estimation of bacterial and fungal cells was made on unspecific culture agar media (TSA and SDA, respectively) in single-species consortia. For microbial isolation in polymicrobial consortia, specific solid growth media were employed. *Pseudomonas* isolation agar (PIA, Sigma) was used to specifically select *P. aeruginosa* when in combination with *S. aureus*, *S. epidermidis*, and *K. pneumoniae*, while TSA supplemented with 10 mg/L of amphotericin B (Sigma-Aldrich) was used to isolate *P. aeruginosa* while suppressing *C. albicans* growth. In turn, *C. albicans* was grown in SDA supplemented with 30 mg/L of gentamicin (Nzytech) to suppress*P. aeruginosa* growth. *S. aureus* and *S. epidermidis* were both differentially selected on Mannitol salt agar (MSA, Liofilchem) and *K. pneumoniae* on Klebsiella ChromoSelect Selective Agar Base (Sigma-Aldrich). Unlike for the other consortia, no selective medium for*A. baumannii* was found, so the total number of adhered cells in TSA was compared with the growth of *P. aeruginosa* only in PIA (allowing to infer about the differential amount of*A. baumannii* cells in consortia).

### 2.8 Live/Dead Staining Applied to Triple-Species Consortia Developed on Modified PVC Surfaces

Antibiofilm performance of CIP/CHX-modified (vs unmodified) PVC surfaces was also qualitatively evaluated through the LIVE/DEAD BacLight Bacterial Viability Kit (Molecular Probes, Leiden, The Netherlands) for the more complex (triple-species biofilm) scenario, thus reflecting a real-like VAP-associated polymicrobial infection. The viability of*P. aeruginosa* single-species biofilms developed on uncoated and coated PVC surfaces was also investigated for comparison purposes. After biofilm formation and washing, the samples were stained for 20–30 min in the dark with a mixture of SYTO 9 (10 μM) and propidium iodide (PI, 60 μM) prepared in PBS solution, as recommended by the manufacturer. For microscopic visualization, an Olympus BX51 fluorescence microscope (Perafita, Portugal) equipped with excitation filters of 470–490 nm in combination with 530–550 nm was used.

### 2.9 Interpretation of Culture-Based Antibiofilm Performance Outcomes

The CFU outcomes from the antimicrobial performance resulting from the co-immobilization of CHX and CIP were classified as “synergism” or “facilitation” according to a previously reported methodology.^26^ An outcome was classified as “synergism” when the coating with combined compounds was able to prevent the adhesion of a greater fraction of microorganisms than expected if the compounds were acting independently. The term “facilitation” was used to classify an outcome in which the coating with combined compounds was better than the best of the single compounds but not better than if the antimicrobials were acting independently. For their calculation, synergism was considered when the equation Log_10_ (*S*_C_) – Log_10_ (*S*_CHX_) – Log_10_ (*S*_CIP_) + Log_10_ (*S*_MIX_) < 0 was valid. In turn, facilitation was considered when both equations Log_10_ (*S*_MIX_) – Log_10_ (*S*_CHX_) < 0 and Log_10_ (*S*_MIX_) – Log_10_ (*S*_CIP_) < 0 were valid. In these equations, *S*_C_ refers to the microbial density obtained in the control (unmodified PVC surfaces) and *S*_CHX_, *S*_CIP_, and *S*_MIX_ refer to the surviving cell density after being in contact with surfaces functionalized with CIP, CHX, or both antimicrobials combined (CIP/CHX), respectively.

### 2.10 Statistical Analysis

Statistical analysis and graphs were performed using the GraphPad Prism software package (GraphPad Software version 8.2.0). Means and standard deviations (SDs) were calculated for all experimental conditions tested. Statistical analysis was carried out by two-way analysis of variance (ANOVA) in the case of the antimicrobial performance against dual and triple species and one-way ANOVA for the remaining assays, followed by Tukey’s multiple comparisons, and *p*-values < 0.05 were considered significant. At least, three independent experiments in triplicate were performed for all experiments.

## 3 Results

### 3.1 pDA-Assisted Immobilization of CIP and/or CHX on PVC

To impart the surfaces of PVC with antimicrobial features, a previously reported one-step pDA-assisted approach for the immobilization of CHX on stainless steel was followed,^22^ now being extended for CIP as well. In this approach, compounds were immobilized by simply immersing PVC substrates in a solution of dopamine dissolved together with CHX or CIP, as depicted in Scheme 1. This method involves the self-polymerization of dopamine monomers resulting in the formation of a thin pDA film, alongside the binding of antimicrobial compounds throughout the pDA layer.^36^ No definite structure model for pDA exists. Dopamine polymerization, despite being quite simple and facile, produces complex redox reactions and a series of intermediates, generating diverse models proposing pDA formation/structure. As recently reviewed, some studies evidence covalently linked aromatic repeat units forming the pDA structure, which contrast with others reporting only supramolecular (noncovalent) bonding. On the other hand, a few models have suggested a mixture of covalent and noncovalent bonding interactions in pDA.^27^ The coexistence of catechol and amine functional groups has been, however, largely accepted.^28^ As so, a simplified mixed catechol/quinone form of pDA is represented in Scheme 1. The lack of an unambiguous pDA structure and clear mechanistic studies driving its formation make the understanding of its interaction with other molecules very complex and speculative. Due to the very nature of the functionalization process, involving the immersion of samples in antimicrobial solutions for long periods (∼18 h), it is mostly expected that favorable physical (noncovalent) adsorption/interaction occurs either by interference in the network of hydrogen (H) bonding between chains or involving π–π stacking, van der Waals forces, and hydrophobic interactions.^29,30^ Therefore, this approach will lead to the development of a release system.

A quick qualitative inspection of the contact antimicrobial and release abilities of CIP and CHX on the modified PVC surfaces at 24 h upon immobilization (Table S1) demonstrated that CHX and CIP could retain dose-dependent antimicrobial/release activities against major VAP-related species, providing evidence of the successfulness of the employed one-step pDA-assisted immobilization approach.

### 3.2 Physicochemical Characterization of CIP and/or CHX-Modified Surfaces

#### 3.2.1 Surface Morphology

**Figure 1:**
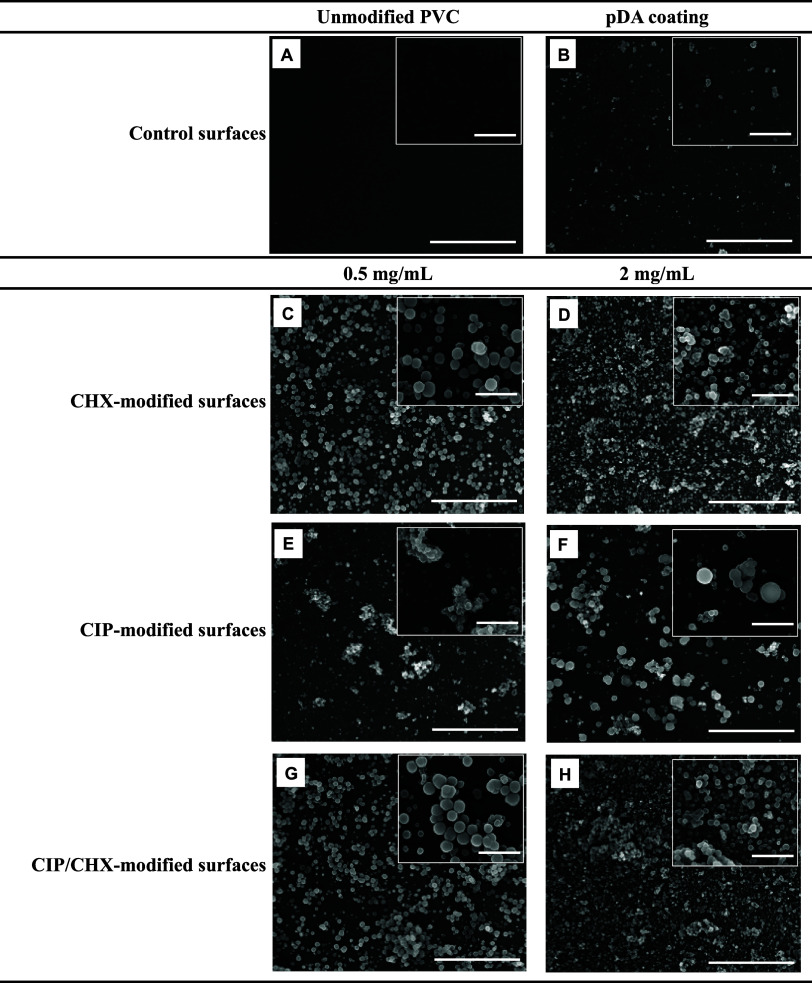
Surface morphology obtained by SEM: (A) unmodified PVC surface, (B) pDA-coated surface, (C) PVC surface modified with CHX at 0.5 mg/mL or with (D) CHX at 2 mg/mL, (E) CIP at 0.5 mg/mL, (F) CIP at 2 mg/mL, (G) CIP/CHX at 0.5 mg/mL, and (H) CIP/CHX at 2 mg/mL. The scale bars in the insets and respective images indicate 2 and 10 μm, respectively.

The surface morphology of PVC surfaces modified with CIP, CHX, and even with both combined (CIP/CHX) was characterized by SEM (Figure 1).

Bare PVC, that is, unmodified PVC surface (Figure 1A), exhibited a smooth morphology with some micropores, likely attributed to the evaporation of solvent during its production.^31^ The pDA film formation on PVC surfaces (Figure 1B) has been suggested by the absence of micropores throughout the surface and the presence of self-polymerized pDA particles resulting from the bulk solution,^32^ thus suggesting the existence of a supramolecular pDA structure.^33^ Dopamine polymerization in the presence of CHX and/or CIP resulted in altered morphologies compared to bare PVC but preserved the structure (in clusters/microspheres) of pDA-coated surfaces, suggesting the interaction of CIP and CHX in the physical self-assembling of pDA. In general, one-step immobilization of both compounds, either alone or combined, yielded surfaces with a more homogeneous appearance, with clusters more evenly distributed throughout the surfaces (Figure 1C–H). A more complete coverage of the surface was observed for CHX (Figure 1C,D) when compared to surfaces functionalized with CIP alone (Figure 1E,F). Increasing the initial concentration of the compound used for PVC functionalization promoted an apparent increase in the number of agglomerates (Figure 1D,F vs C,E, respectively). Co-immobilization of CHX and CIP (CIP/CHX; Figure 1G,H) yielded surfaces with morphology and coverage similar to those incorporating CHX alone.

#### 3.2.2 Surface Roughness and Wettability

**Figure 2:**
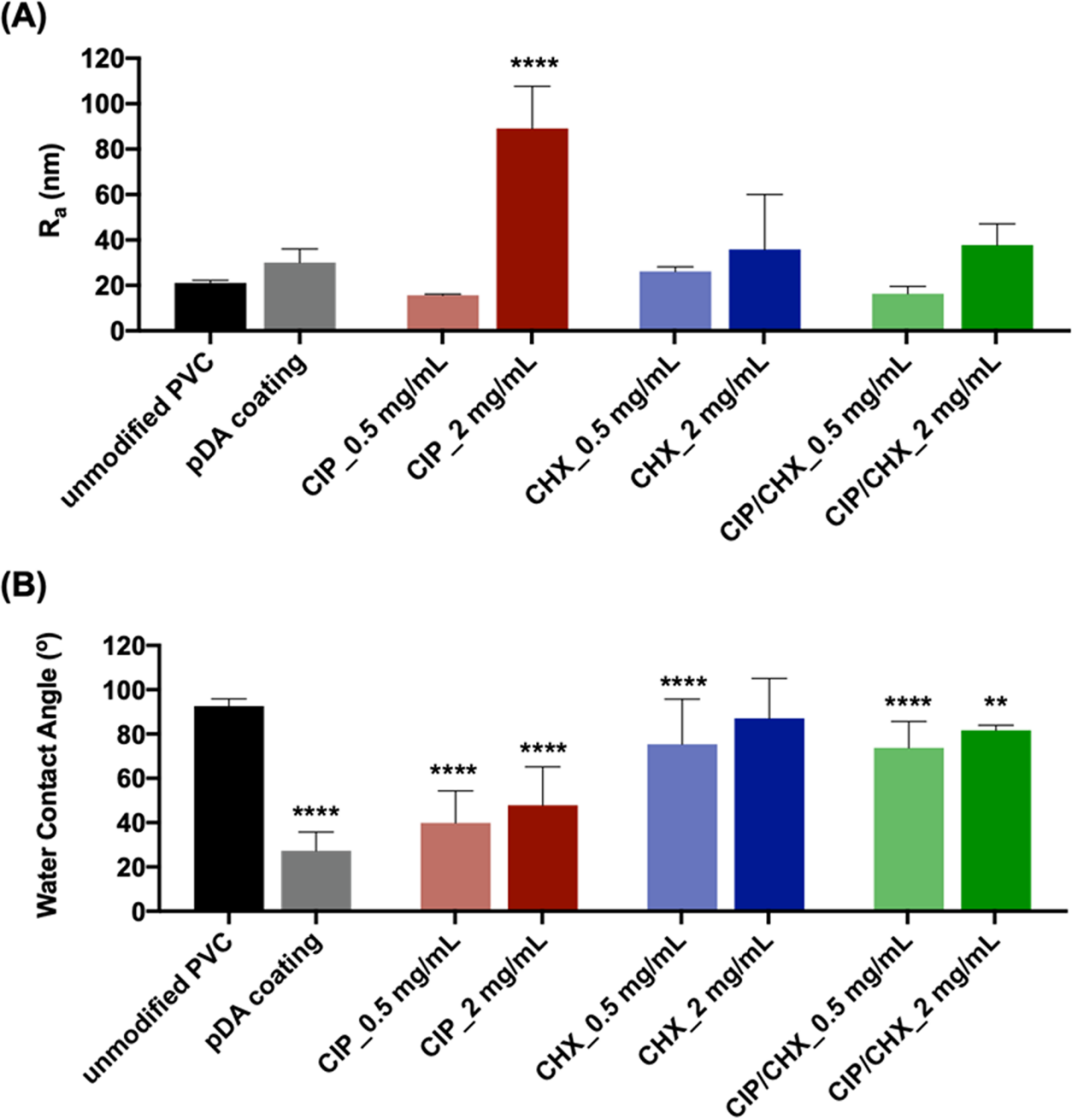
Surface roughness and wettability. (A) Average roughness and (B) static water contact angles of PVC surfaces before and after immobilization with CIP, CHX, or CIP/CHX at concentrations of 0.5 and 2 mg/mL. Unmodified PVC and pDA-coated surfaces were used for comparison. The results are shown as mean ± SDs. (****) *p* < 0.0001, (**) *p* < 0.01,vs unmodified PVC, one-way ANOVA, Tukey'′s multiple comparison test.

Changes in surface roughness and wettability caused by the different immobilization approaches were investigated by AFM and by measuring the static water contact angles, respectively (Figure 2).

The average surface roughness, expressed as *R*_A_ (Figure 2A), was determined from the pictures obtained from AFM analysis (Figure S1 in the Supporting Information). Overall, results showed that pDA coating did not introduce significant changes in the PVC surface’s roughness as well as further functionalization with CIP and/or CHX. Although not significantly different in most cases, the results suggest an increase in the modified surfaces’ roughness as the concentration of immobilized compounds increases. An exception was observed for PVC surfaces functionalized with CIP at 2 mg/mL, which displayed a significant increase (4-fold higher) in that parameter. The effect of antimicrobials on the roughness of surfaces may rely on the agent itself and respective concentration and the surfaces involved. At higher concentrations, CIP molecules can self-assemble and form aggregates due to the aromatic rings in its structure.^34^ In turn, the CHX immobilization on the pDA film caused a modest increment of surface roughness, as illustrated in Figure 2A. It has been recently reported that the mechanical properties of pDA films can be coordinated by the addition of new molecules.^30^ Previous findings demonstrated the higher surface roughness on CHX coatings due to the occurrence of CHX aggregates and several protrusions on the grafted pDA layer.^35^ Although specific research would be needed to provide detailed insights into the interaction between CHX and pDA, a reasonable justification for the less noticed roughness reduction (as compared to CIP-coated surfaces) shown in Figure 2A is the ability of CHX to form H-bonds with pDA chains while using the bridge of saturated carbons as a spacer. This may cause the pDA polymeric mesh to be less compact and the CIP molecules remain in this mesh, instead of disturbing the H-bond network typical of pDA. This eventually explains the lower roughness observed for CIP/CHX-modified surfaces as compared to surfaces modified with CIP alone.

Water contact angles (Figure 2B) evidenced a hydrophobic character for unmodified PVC surfaces (92.6° ± 3.2°). Dopamine polymerization imparted the PVC surfaces with hydrophilic properties, as evidenced by the significant decrease of the water contact angle to below 90° (27.3° ± 8.5°), a well-established observation found on other materials functionalized with pDA.^14,15^ Concerning modified surfaces, the incorporation of CHX during dopamine polymerization resulted in an increase in the water contact angle (75.3° ± 20.4° and 87.1° ± 18.0°), turning surfaces into more hydrophobic features. On the other hand, PVC functionalization with CIP did not greatly interfere with the hydrophilic properties imparted by pDA alone. Co-immobilization of CHX and CIP (CIP/CHX) yielded surfaces with wettability similar to the CHX-immobilized ones. Overall, increasing the initial concentration of the immobilized compounds, when alone or combined, slightly increased the hydrophobic properties of the modified surfaces.

#### 3.2.3 Surface Molecular Composition

**Figure 3:**
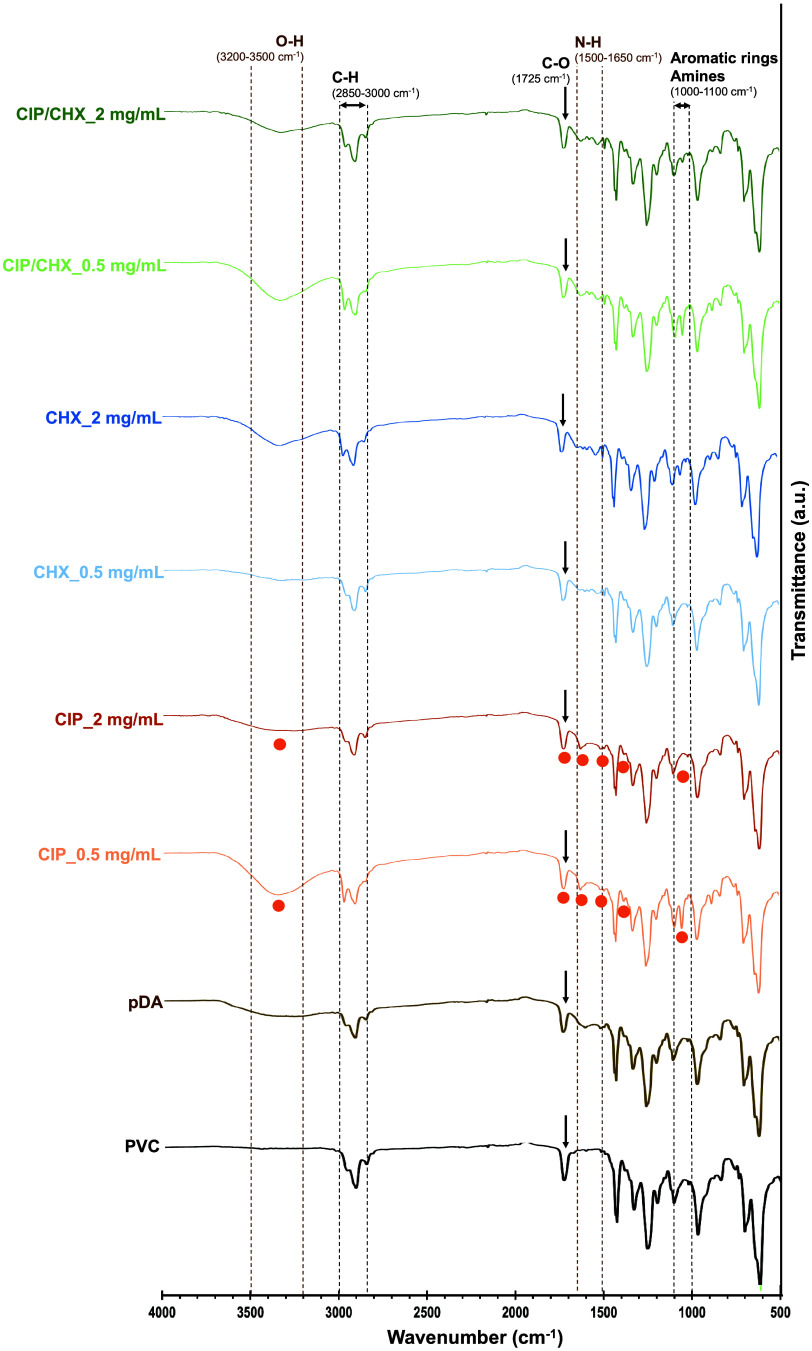
ATR-FTIR spectra of PVC surfaces before and after immobilization with CIP, CHX, or CIP/CHX at concentrations of 0.5 and 2 mg/mL.

The chemical characterization of unmodified-, pDA-, and CIP and/or CHX-modified PVC surfaces was carried out by a semiquantitative ATR-FTIR analysis in a 500–4000 cm^–1^ wavenumber, where the obtained spectra are depicted in Figure 3.

FTIR results exhibited the successful oxidative polymerization of dopamine resulting in the pDA deposition on PVC surfaces, as supported by the appearance of a large, broad peak spanning 3200–3500 cm^–1^, consistent with the presence of catechol hydroxyl structures as well as water. Another characteristic absorption peak in pDA was discernible between 1500 and 1650 cm^–1^ due to N–H stretching vibrations in the primary amine.^36^ In addition, the presence of a band at 1725 cm^–1^ (indicated by the black arrows) revealed the presence of carbonyl (C=O) groups, evidencing the formation of the quinone content and substantiating a catechol/quinone chemical equilibrium on the pDA structure, as described earlier. The presence of a peak at the same wavenumber on PVC surfaces may be related to the type of polymeric material or an effect of UV sterilization^37^ causing PVC photodegradation and subsequent formation of short polymeric chains containing C=O and –CH=CH– moieties.^38^

The successful loading of CIP and CHX was corroborated by the retention of the characteristic pDA absorption peaks on the modified surfaces (highlighted by the shaded zones in Figure 3). In addition, the peaks denoted by orange circles at 3350 cm^–1^ (–OH stretch), 1725 cm^–1^ (–CO stretch), 1627 cm^–1^ (C=O vibration), 1491 cm^–1^ (C–H stretch), 1360 cm^–1^ (aromatic C=C), and around 1050 cm^–1^ (C–F stretching) are characteristic of CIP spectra.^38^ Alongside, absorption peaks between 1050 and 1100 cm^–1^ related to the presence of aromatic rings and primary and secondary amide bonds,^35^ as well as bands observed at wavelengths between 2850 and 3000 cm^–1^ corresponding to alkane C–H-bonds (a strong indicator of the long hydrocarbon chain of CHX),^35^ were observed in all analyzed PVC surfaces. Overall, no linear and direct correlation between the compound concentration and band intensity was found in the analyzed FTIR spectra. A reliable way to find a precise connection between both parameters would be through calibration procedures.

#### 3.2.4 CHX and CIP Release Profiles

**Figure 4:**
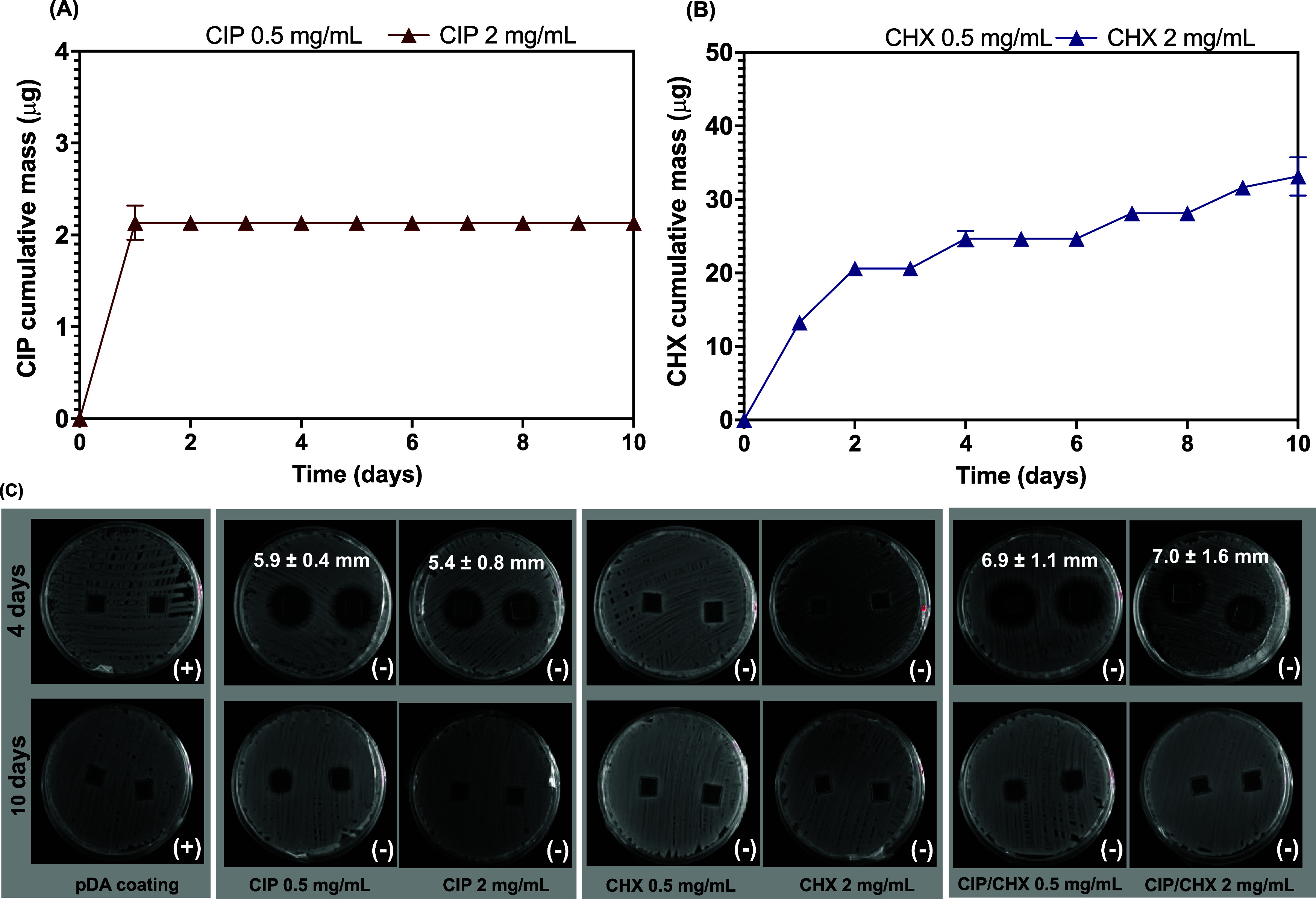
Antimicrobials released from coated surfaces. (A) Cumulative release of CIP and (B) of CHX from PVC surfaces functionalized with each compound at 0.5 and 2 mg/mL. Panel (C) depicts the outcomes from the contact antimicrobial activity and the release of CIP or CHX-immobilized alone, and CIP/CHX co-immobilized on PVC surfaces at 0.5 and 2 mg/mL, at days 4 and 10 against *K. pneumoniae*. Bacterial growth after 24 h contact with modified surfaces is indicated at the bottom right of each image, where “+” is indicative of visible bacterial growth and “–” means no visible growth observed. The release of antimicrobials on solid agar was evaluated by the presence or absence of an inhibition zone. The average length of inhibition zones was determined using ImageJ and is presented in mm. pDA-coated surfaces were used for comparison.

The released amount of CHX and CIP from functionalized PVC surfaces was monitored each day for up to 10 days by measuring the absorbance by UV–vis and further determining the released amount of each compound through previously established calibration curves. The profiles obtained for CIP and CHX cumulative released masses are illustrated in Figure 4.

Overall, there was an initial burst release of CIP immobilized at both concentrations, which was observed in the first 24 h after its immobilization on the PVC surfaces (Figure 4A). During the following days, CIP could not be detected (no CIP mass added). A comparable trend was noted for PVC surfaces functionalized with a higher CIP concentration (2 mg/mL), however, showing an enhanced released amount of CIP in comparison to those surfaces immobilized with a lower CIP concentration. A significantly higher cumulative mass was found for CHX (Figure 4B), as compared to CIP, with cumulative mass estimations of nearly 40 μg after 10 days of release. As for CIP, a rampant increase in the CHX amount was found for the early 24 h, followed by a slower release of the antimicrobial in the remaining days. Initially, the amount of liberated CHX increased as the immobilized CHX concentration also increased (<4 h). From then on, PVC surfaces functionalized with a lower CHX concentration (0.5 mg/mL) started to release higher CHX amounts than those immobilized with CHX at 2 mg/mL. Our findings showed that, despite the high initial antimicrobial concentration (0.5 or 2 mg/mL), only a small amount of CIP and CHX could be detected/estimated after 10 days of release. This leads us to suppose that some stronger (eventually covalent) coupling may also occur in addition to noncovalent reversible bonding of both antimicrobials to the pDA polymer (as described in Section 3.1), potentially leading to sustained release over time. Certain H-bonding interactions are possible to occur, e.g., via piperazine ring in CIP or the guanidine-containing group in CHX and the carbonyl groups of the catechol moieties of pDA.^39,40^

As stated above, the apparent CIP depletion in the PBS supernatant for the period after 24 h of release was likely attributed to a low-limit detection (0.54 μg/mL, while 1.05 μg/mL was obtained for CHX). To support this, the presence of released CIP, alone and combined with CHX, was further investigated on days 4 and 10 based on direct contact antimicrobial activity and release approaches, as demonstrated in panel C of Figure 4.*K. pneumoniae*, shown as the most susceptible species to both CIP and CHX in broth microdilution testing (Table S2 of the Supporting Information), was used so that lower antimicrobial concentrations could be detected. At day 4, all CIP-modified surfaces exhibited contact antimicrobial activity and formed inhibition zones against *K. pneumoniae*. Such results still evidenced immobilization of CIP, either alone or combined, on the PVC surfaces but in small quantities, likely not detected by UV spectroscopy. Additionally, no significant differences were found in the length of inhibition zones obtained for CIP immobilization at 0.5 or 2 mg/mL, an indication that after 4 days, the release was similar in both conditions. The presence of CHX in the supernatant was also appraised by means of contact antimicrobial activity and release assets. As expected, the absence of an inhibition zone may be a reflection of its low release concentration on day 4 (Figure 4B), which was found below its minimum microbiocidal concentration (MMC; Table S2). A higher inhibition zone obtained for the CIP/CHX-modified surfaces, as compared to surfaces modified with CIP only, strongly suggests a higher antimicrobial release, which can be attributed to the presence of CHX as well. After 10 days of release, no inhibition zone was observed for any modified surface, but all CIP, CHX, and CIP/CHX-modified surfaces retained their contact antimicrobial activity (as indicated by the symbol “(−)” at the bottom right of the images), evidence of an effective antimicrobial immobilization and prolonged antimicrobial activity.

### 3.3 Cytotoxicity against A549 Lung Epithelial Cells

**Figure 5:**
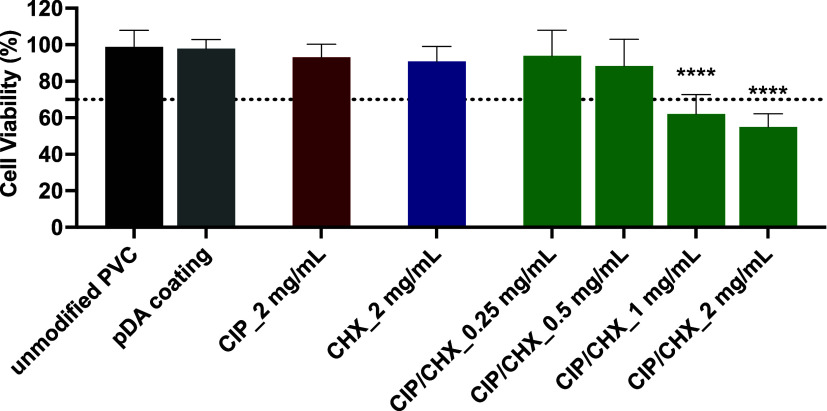
Cell cytotoxicity. Viability of A549 lung epithelial cells after indirect contact with PVC surfaces functionalized with CIP or CHX at the highest concentration tested (2 mg/mL) and with CIP/CHX at 2 mg/mL and lower concentrations (1 mg/mL, 0.5 mg/mL, and 0.25 mg/mL). Unmodified PVC and pDA-coated surfaces were used for comparison. A threshold for cell toxicity of 70% viability was used, which is indicated by a dotted line. The results are shown as mean ± SDs. (****) *p* < 0.0001, vs unmodified PVC, one-way ANOVA, Tukey’s multiple comparison test.

Aiming at evaluating the toxicity of PVC-modified surfaces to lung epithelial cells, PVC surfaces immobilized with CIP and CHX, alone or combined, were placed in contact with a DMEM culture medium, which was further exposed to A549 cells. The cell viability (expressed as a percentage) of indirectly contacting PVC surfaces immobilized with CIP and/or CHX is presented in Figure 5.

A large percentage of A549 viable cells was obtained when they were in indirect contact with unmodified PVC before and after pDA coating (Figure 5). Further functionalization of PVC surfaces with CIP or CHX at 2 mg/mL also produced no significant cytotoxic effect on A549 eukaryotic cells, as compared with unmodified surfaces. In turn, CIP/CHX co-immobilization with the highest antimicrobial concentration tested (2 mg/mL) on a PVC surface resulted in a decline in the number of viable cells higher than 30%, evidence of toxicity (<70% cell viability) toward eukaryotic cells. In a way to reduce cytotoxicity, doses lower than 2 mg/mL were tested for co-immobilization, and the viability of A549 cells exposed to DMEM medium, which was put before in contact with these modified surfaces, was also appraised. Cytotoxicity was found to be dose-dependent, with the highest antimicrobial concentrations (2 and 1 mg/mL) causing toxic effects on A549 cells, contrariwise to CIP/CHX doses lesser than 0.5 mg/L, which did not affect cell viability. Based on these findings, only modified PVC surfaces with proven biocompatibility proceeded to the next antibiofilm assays.

### 3.4 Efficacy of Modified PVC Surfaces in Preventing Single-Species Biofilms

**Figure 6:**
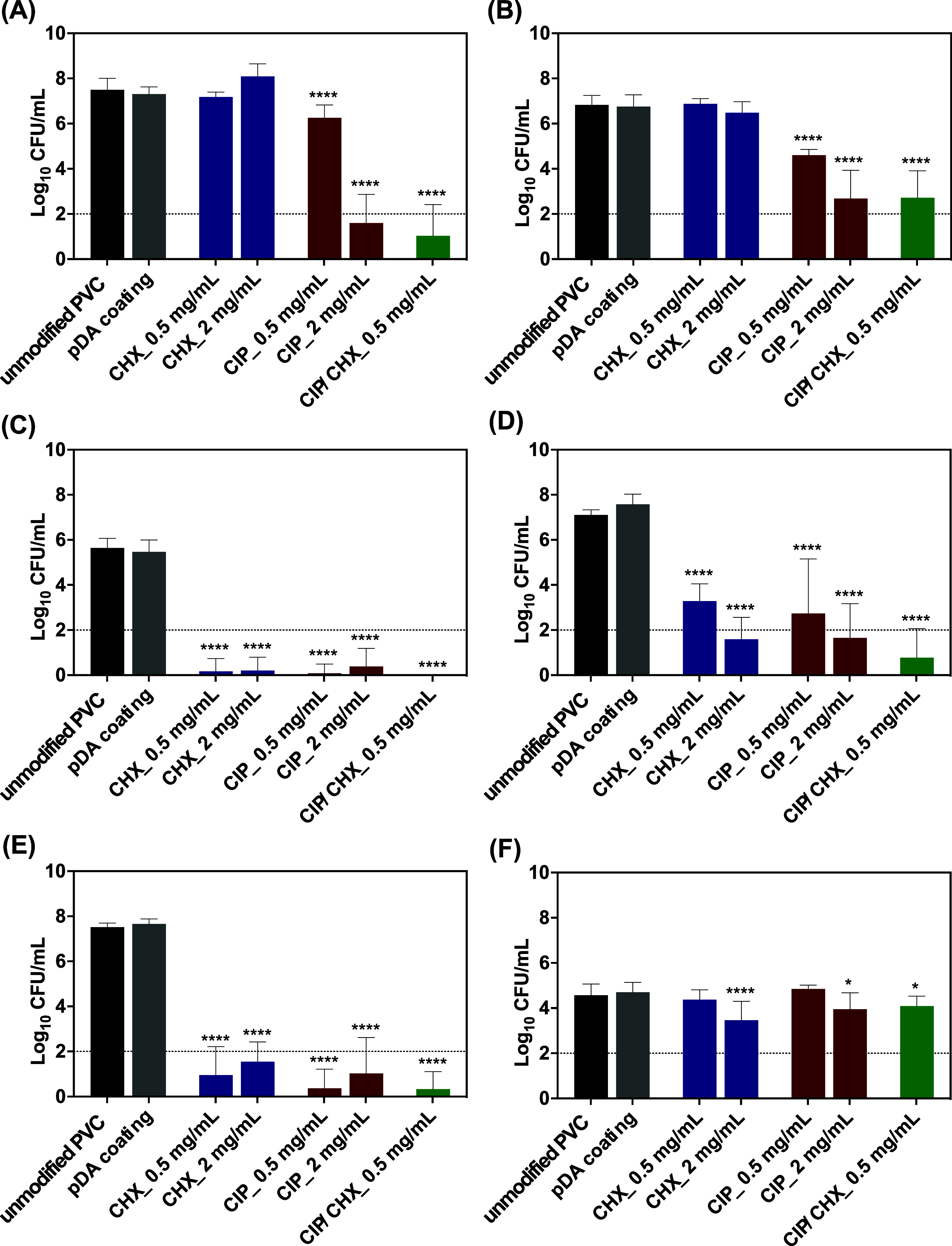
Efficacy against single-species biofilms. PVC surfaces modified with CIP and CHX were challenged with single-species biofilms formed by*P. aeruginosa* (A),*A. baumannii* (B),*K. pneumoniae* (C),*S. aureus* (D),*S. epidermidis* (E), and*C. albicans* (F). Unmodified PVC and pDA-coated surfaces were used for comparison. The threshold value marked by the dotted line represents the detection limit of CFU counting. The results are shown as mean ± SDs. (****) *p* < 0.0001, (*) *p* < 0.05 vs unmodified PVC, one-way ANOVA, Tukey’s multiple comparison test. (****) *p* < 0.0001, vs unmodified PVC, one-way ANOVA, Tukey’s multiple comparison test.

As microbial adhesion is the preceding step of biofilm formation, which represents an early and recurrent event in intubated patients,^5,41,42^ the next stage was to appraise the effectiveness of the modified PVC surfaces in inhibiting microbial adhesion and so preventing the biofilm development by microorganisms commonly associated with VAP, namely,*P. aeruginosa*,*A. baumannii*,*K. pneumoniae*,*S. aureus*,*S. epidermidis*, and*C. albicans*. Thus, PVC surfaces modified with CIP and/or CHX that displayed no cytotoxicity (0.5 and 2 mg/mL) were challenged for 24 h with microbial suspensions of each aforementioned species, and the number of cultivable cells that adhered to the surfaces was enumerated after detachment (Figure 6).

Results show that the ability to form biofilms on bare/unmodified PVC surfaces relied on the microbial species, with most biofilms achieving high cell densities varying from 6.83 and 7.52 Log_10_ CFU/mL after 24 h. Lower cell concentrations, however, were found for*K. pneumoniae* (Figure 6C) and*C. albicans* (Figure 6F), reaching 5.64 and 4.57 Log_10_ CFU/mL on average, respectively. As anticipated, the pDA film on PVC did not affect the adhesion of any microbial species, as evidenced by the similar number of adhered cells found compared to that of the unmodified PVC control.

Functionalization with CHX and/or CIP rendered the PVC surfaces with wide-ranging antibiofilm activities. CHX-functionalized surfaces were not enabled to prevent biofilm formation by*P. aeruginosa* (Figure 6A) and*A. baumannii* (Figure 6B), regardless of the CHX initial concentration applied. However, significant prevention of microbial colonization was found for the remaining biofilm populations (Figure 6, parts C–F). A dose-dependent decrease in cell attachment was observed not only for*S. aureus*, by 3.8 Log_10_ (CHX 0.5 mg/mL) and 5.5 Log_10_ (CHX 2 mg/mL) (Figure 6D), but also for*C. albicans* biofilms, the formation of which was modestly inhibited (∼1.1 Log_10_) when CHX 2 mg/mL was immobilized on PVC surfaces (Figure 6F).

As for CHX, a great ability to inhibit biofilm formation and, in most cases, a dose-dependent effect was observed for CIP-functionalized PVC surfaces. For instance,*P. aeruginosa* biofilm growth was prevented from 6.26 to 1.60 Log_10_ CFU/mL (Figure 6A),*A. baumannii* from 4.60 to 2.68 Log_10_ CFU/mL, and*S. aureus* from 2.73 to 1.65 Log_10_ CFU/mL (Figure 6D) when grown on PVC immobilized with CIP at 2 mg/mL compared to CIP at 0.5 mg/L. CIP immobilization at 2 mg/mL still caused a slight inhibition (<1 Log_10_) in the number of fungal biofilm cells (Figure 6F). The highest inhibitory outcomes were found for biofilms of*K. pneumoniae* (Figure 6C),*S. aureus* (Figure 6D), and*S. epidermidis* (Figure 6E), with the modified surfaces substantially impairing their development (cell numbers were found below thresholds for cultivability).

Interestingly, co-immobilization of CIP and CHX exhibited a significant antibiofilm effect against all consortia investigated. The estimated outcomes of PVC co-immobilization regarding its antibiofilm performance (Table S3 of the Supporting Information) demonstrated a facilitative effect against all microbial species and even a synergic outcome against*P. aeruginosa*,*A. baumannii*, and*C. albicans*. For better understanding, calculations showed higher antibiofilm performance of CIP-CHX co-immobilization than the best of the single compounds acting on all consortia, also enabling prevention of a greater fraction of microbial cells’ adhesion in*P. aeruginosa*,*A. baumannii*, and *C. albicans* biofilm communities than expected if the compounds were acting independently.

### 3.5 Efficacy of Modified PVC Surfaces against Dual-Species Biofilms

**Figure 7:**
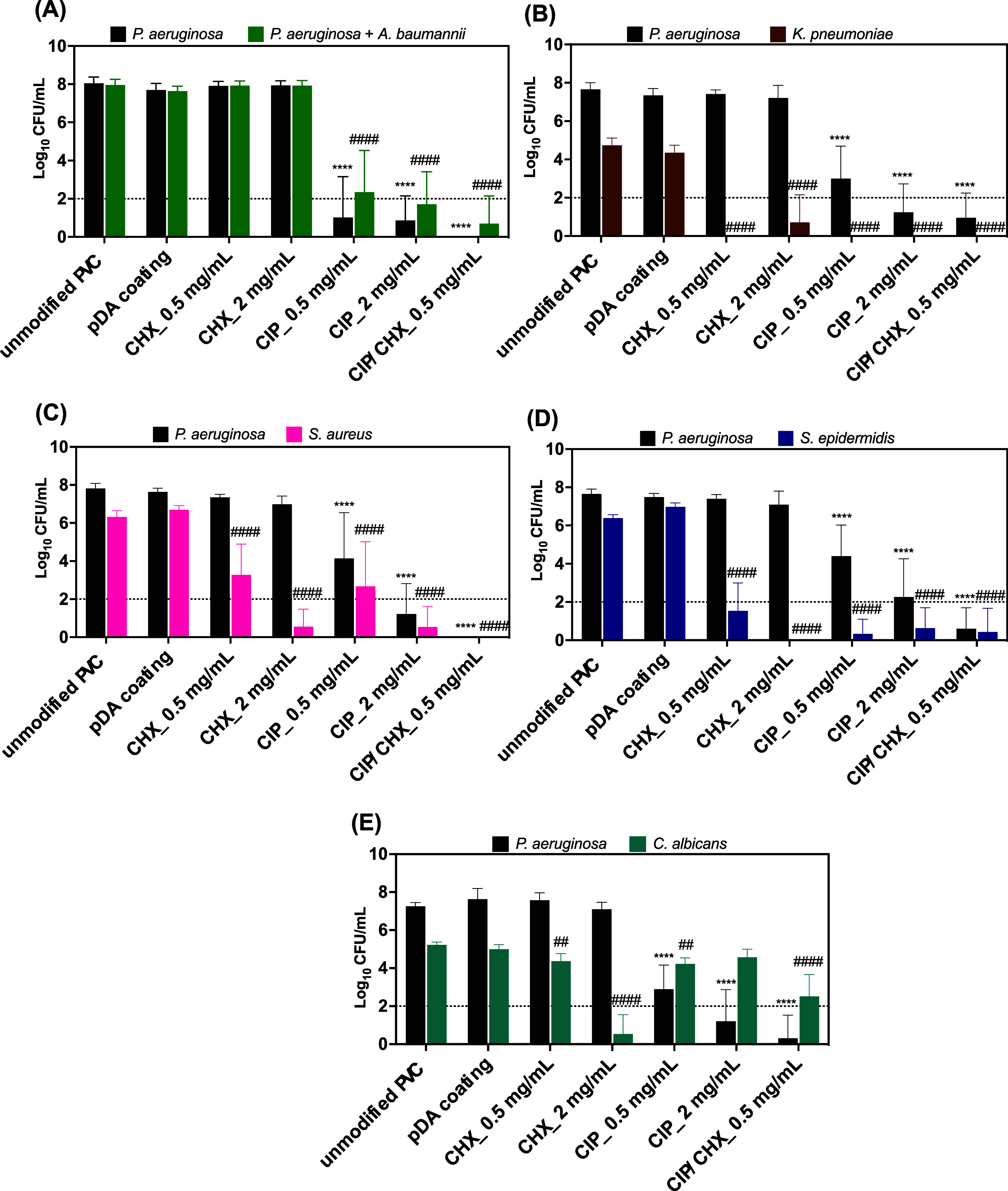
Efficacy against dual-species biofilms. Effect of PVC surfaces before and after pDA-based coating and immobilization of CIP and/or CHX against dual-species biofilms of*P. aeruginosa* and*A. baumannii* (A),*P. aeruginosa* and*K. pneumoniae* (B),*P. aeruginosa* and*S. aureus* (C),*P. aeruginosa* and*S. epidermidis* (D), and*P. aeruginosa* and*C. albicans* (E). Unmodified PVC and pDA-coated surfaces were used for comparison. The threshold value marked by the dotted line represents the detection limit of CFU counting. The results are shown as mean ± SDs. (****) *p* < 0.0001 vs *P. aeruginosa* growth on unmodified PVC and (^##^) *p* < 0.01 or (^####^) *p* < 0.0001 vs other than *P. aeruginosa* species growth on unmodified PVC, two-way ANOVA, Tukey′s multiple comparison test.

Considering the great prevalence of*P. aeruginosa* on VAP,^8,43^ the PVC surfaces immobilized with CIP and CHX, alone or combined, were then evaluated against the biofilm formation of this species combined with each one of the other tested microbial species (Figure 7).

Overall, dual-species biofilms were mostly governed by*P. aeruginosa*, regardless of the PVC material where the consortia were formed. Biofilms developed on unmodified surfaces (PVC and pDA) presented cell numbers in orders of magnitude similar to those observed for*P. aeruginosa* alone (see Figure 6A), denoting a low influence in*P. aeruginosa* growth of the other microbial species presented in consortia.

CHX-modified surfaces were able to significantly disturb all other than*P. aeruginosa* populations in the consortia (Figure 7A–E), lowering CFU counts to below the detection limit or even leading to full growth prevention of some bacterial species, namely,*K. pneumoniae* (Figure 7B) and*S. epidermidis* (Figure 7D). The absence of a specific medium for the isolation of *A. baumannii* is a limitation of this study, preventing us to infer about the influence of PVC surfaces on*A. baumannii* growth (when in consortia with*P. aeruginosa*). For PVC, pDA, and the coatings with CHX alone, no significant differences were detected between the number of adhered cells found on TSA or the selective*P. aeruginosa* medium, PIA (Figure 7A), which is indicative that*P. aeruginosa* is still likely the most dominant species in these conditions (as ascertained by Figure S2 in the Supporting Information). However, a lack of effectiveness of CHX-modified surfaces against the*A. baumannii* population would be expected similar to the one observed in its single-species biofilms.

When coming to CIP-modified surfaces, drastic dose-dependent inhibitions were estimated for the biofilm populations compared with those developed on unmodified PVC surfaces. Such inhibitions were more pronounced for the bacterial populations in all consortia (Figure 7A–D), as compared with the fungal population (Figure 7E). Concerning*P. aeruginosa* and*A. baumannii* consortia (Figure 7A), equitable CFU numbers were found for each bacterial population within the biofilms grown in CIP-modified surfaces, with PIA estimating about 1.0 Log_10_ and 0.9 Log_10_ CFU/mL of*P. aeruginosa* cells, whereas*A. baumannii* presented ∼1.3 Log_10_ and 0.7 Log_10_ CFU/mL, given by the difference between TSA and PIA counts, respectively (CIP 0.5 mg/mL vs 2 mg/mL). As compared to their single-species biofilms (see Figure 6A,B), the extent of adhesion of both populations in dual-species consortia was notoriously affected by CIP-modified surfaces, preventing*P. aeruginosa* growth by approximately 5.3 Log_10_ and 0.8 Log_10_ and*A. baumannii* by 3.3 Log_10_ and 2.0 Log_10_ (CIP 0.5 mg/mL vs 2 mg/mL). When CIP was immobilized and combined with CHX, they also led to greater inhibitions in*P. aeruginosa* (∼1 Log_10_) and*A. baumannii* (∼2 Log_10_) populations compared to its effect in single-species consortia.

Biofilm formation by*K. pneumoniae* and*S. aureus* on unmodified PVC and pDA surfaces was compromised by the presence of*P. aeruginosa*, as evidenced by ca. 1 Log_10_ inhibition in the number of biofilm cells compared to their single-species biofilm (Figure 7B,C vs Figure 6C,D, respectively). Further PVC functionalization with CIP and CHX, alone or combined, was able to substantially prevent colonization of both species (Figure 7B,C), even fully preventing the*K. pneumoniae* biofilm population, a result comparable to that observed for its single-species biofilm (Figure 7B vs Figure 6C). Similar to other bacterial species,*S. epidermidis* biofilm formation on untreated surfaces was impaired by the presence of*P. aeruginosa*, as evidenced by the inhibition of approximately 1 and 0.7 Log_10_ for unmodified PVC and pDA surfaces, respectively. However, it should be taken into account that some of this inhibition could be associated with its growth in the selective media (Figure S2 in the Supporting Information). All of the CIP- and/or CHX-modified surfaces caused a significant prevention of the number of*S. epidermidis* cells in the dual-species consortia (Figure 7D), an antimicrobial performance similar to the one obtained for its single-species adhesion (see Figure 6E).

Contrariwise to the consortia involving only bacterial species, biofilms of*P. aeruginosa* and*C. albicans* were not always dominated by*P. aeruginosa* (Figure 7E). As illustrated, a slight increase in the number of fungal cells was found of about 0.7 Log_10_ and 0.3 Log_10_ adhered to PVC and pDA, respectively, as compared to single-species biofilms (Figure 6F). Despite this increase in fungal ability to adhere to PVC and pDA surfaces, coating strategies proved to be more efficient in preventing its adhesion. The best approach was PVC functionalized with CHX at the highest concentration (2 mg/mL), able to inhibit 4.7 Log_10_ (as opposed to the 1.1 Log_10_ inhibition achieved for its single-species adhesion). Co-immobilization of CIP and CHX was also able to significantly impair the adhesion of*C. albicans* of ca. 2.7 Log_10_. The antimicrobial performance of this coating strategy against*P. aeruginosa* in this consortium was similar to or better than the one obtained for its single-species adhesion.

Co-immobilization of CIP and CHX proved to result in a synergic effect against*P. aeruginosa* for all consortia investigated, according to the calculations in Table S4 of the Supporting Information. A similar synergic effect endured against*C. albicans* and a facilitative one against all other microbial species in consortium with*P. aeruginosa*.

### 3.6 Efficacy of Modified PVC Surfaces against Triple-Species Biofilms

**Figure 8:**
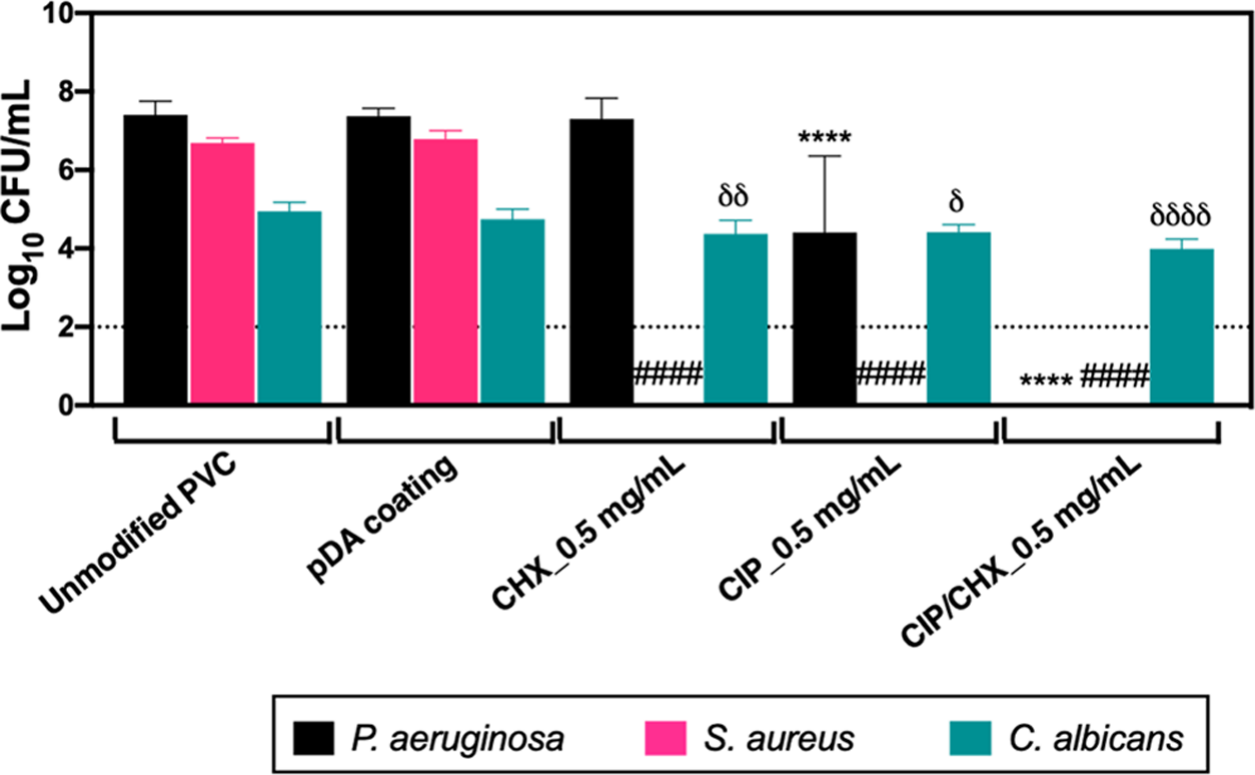
Efficacy against triple-species biofilms. Effect of PVC surfaces before and after pDA-based coating and immobilization of CIP and/or CHX against triple-species biofilms of*P. aeruginosa*,*S. aureus*, and*C. albicans*. Unmodified PVC and pDA-coated surfaces were used for comparison. The threshold value marked by the dotted line represents the detection limit of CFU counting. The results are shown as mean ± SDs. (****) *p* < 0.0001 vs *P. aeruginosa* growth on unmodified PVC; (^####^) *p* < 0.0001 vs *S. aureus* species growth on unmodified PVC; and (^∂^) *p* < 0.05, (^∂∂^) *p* < 0.01, and (^∂∂∂∂^) *p* < 0.0001 vs *C. albicans* species growth on unmodified PVC two-way ANOVA, Tukey′s multiple comparison test.

To better mimic the true polymicrobial (that often includes interkingdom) nature of VAP infections and evaluate the dual-drug CIP/CHX coating strategy’s success in a more complex (and eventually worsening) scenario, its effectiveness in preventing the development of a triple-species biofilm was investigated. Biofilms comprised of not only three populations involving Gram-negative (*P. aeruginosa*) and Gram-positive (*S. aureus*) bacteria but also fungal species (*C. albicans*) were grown on CIP/CHX-modified surfaces, and their CFU number was estimated after 24 h (Figure 8).

The ability of*P. aeruginosa* or*C. albicans*, grown in triple consortia, to adhere to control unmodified PVC and pDA-coated surfaces, was not compromised by the presence of other microbial species in the biofilm, as compared to their single-species adhesion (see Figure 6A,F). On the other hand, minor growth inhibitions (<1 Log_10_) occurred for*S. aureus* populations (Figure 8 vs Figure 6D), in a similar way to when it was combined with*P. aeruginosa* in dual-species consortia (see Figure 7D). All modified surfaces, immobilized with CIP and CHX alone or combined, contributed significantly to impairing the adhesion of*S. aureus*. In turn, PVC functionalized with CHX alone had no effect against the*P. aeruginosa* population, as expected, but its growth was considerably disturbed when biofilms were developed on CIP- and CIP/CHX-modified surfaces, even resulting in pseudomonal prevention for CIP/CHX-modified surfaces (Figure 8). However, the antibiofilm effect of all coatings was not demonstrated for*C. albicans* populations, as attained for the bacterial populations in the consortia. Furthermore, the modified surfaces had a more modest antifungal effect against triple-species biofilms when compared to that attained in dual-species*P. aeruginosa*/*C. albicans* biofilms (Figure 7E). Even so, the dual-drug CIP/CHX coating was promising in substantially affecting bacterial populations inside the consortia and even modestly preventing *C. albicans* cell numbers by about 1 Log_10_ (compared to unmodified PVC).

### 3.7 Viability of Biofilms Formed on Uncoated vs CIP/CHX-Coated Surfaces

**Figure 9:**
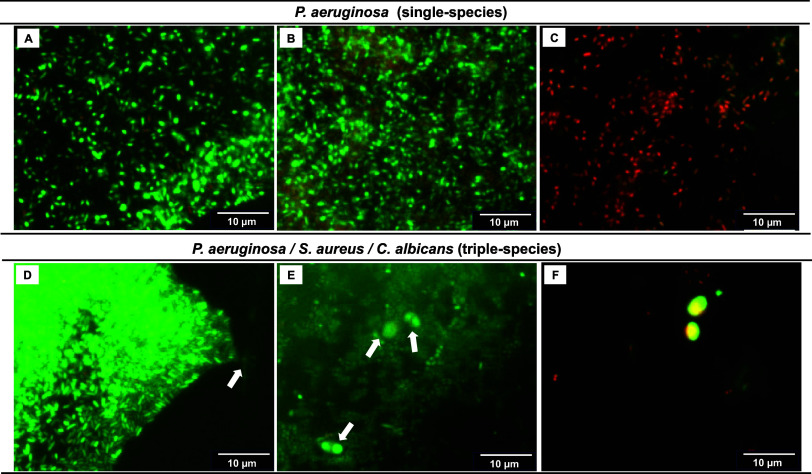
Biofilm viability. On the top row,*P. aeruginosa* single-species biofilms developed on uncoated PVC (A), pDA-coated PVC (B), and CIP/CHX-modified surfaces (C). On the bottom row,*P. aeruginosa*,*S. aureus*, and*C. albicans* triple-species biofilms developed on uncoated PVC (D), pDA-coated PVC (E), and CIP/CHX-modified surfaces (F). Biofilms were stained with LIVE/DEAD BacLight Bacterial Viability system, where live cells were stained with the green fluorescent dye SYTO 9, and compromised/damaged cells were labeled in red by the PI stain.

For a more comprehensive understanding of the potential of our coating strategy to prevent biofilm adhesion in a real-like scenario of polymicrobial infection, we conducted a qualitative analysis on the viability of the triple-species biofilms formed on uncoated PVC, pDA-coated PVC, and CIP/CHX-modified surfaces using the LIVE/DEAD BacLight Bacterial Viability Kit, allowing us to discriminate among live and dead cells based on membrane integrity.^44^ For comparison, the viability of*P. aeruginosa* single-species biofilms was also investigated (Figure 9). Apparently, the live/dead assay yielded comparable results to CFU counting. Most*P. aeruginosa* cells in single-species biofilms (Figure 9; top row) developed on uncoated PVC (Figure 9A) and pDA-coated surfaces (Figure 9B) were stained green, indicating that they were mostly viable. By contrast,*P. aeruginosa* biofilms formed on CIP/CHX-modified surfaces showed a sharp decrease in the number of green-labeled viable cells, while a higher proportion of red-labeled compromised cells were notorious (Figure 9C). Similar results were obtained for triple-species biofilms formed by*P. aeruginosa*,*S. aureus*, and*C. albicans* (bottom row). When grown on PVC only (Figure 9D) and on pDA-coated surfaces (Figure 9E), the consortia were mostly stained green, which is therefore indicative of viability. The live/dead assay was consistent with CFU data (Figure 8), where a higher abundance of bacterial cells compared to*C. albicans* was denoted (indicated by arrows as they were sparsely detected and often appeared on different focus plans), confirming that biofilms formed on unmodified surfaces were governed by bacterial populations. However, the adhesion as well as the integrity of these triple consortia was found to be hugely affected when grown on CIP/CHX-coated surfaces (Figure 9F), with the fungal cells still retaining viability and the bacterial cells significantly reducing in number and the red-labeled cells indicating damage. Taking together, CFU and live/dead outcomes denote the potential of the dual-drug coating strategy in substantially preventing biofilm formation and even impairing its viability, especially concerning bacterial communities.

## 4 Discussion

In the last few decades, technology has advanced, although at a slow pace, regarding the development of new active and/or passive materials for improving ETT surfaces.^11^ This relatively slow pace is not attributable to a shortage of new ideas, as recently evidenced, but to the challenge of overcoming fundamental hurdles associated with VAP (e.g., persistent colonization/biofilm formation, mechanical ventilation time, ICU length of stay, VAP occurrence). In addition, the design and implementation of new biomaterials often imply following specific criteria (e.g., antimicrobial activity, safety to use, long-term stability, and surface physicochemical properties) to achieve clinical successfulness. This study, which links up with our previous report showing the potential of the immobilization of CHX using a one-step pDA-functionalization approach in the context of orthopedic devices,^14^ aimed to improve the PVC-ETT design by consistently employing dopamine strategy in the immobilization of CHX and CIP on PVC surfaces.

The use of pDA chemistry, enabled with unique features such as simple functionalization protocol, high biocompatibility, or strong adhesive properties, has attracted considerable attention as one of the simplest and most versatile platforms to functionalize material surfaces with broad potential use in several areas.^45^ Despite the mono-^14^ and multidrug-loaded approaches^22^ that have been developed, pDA-assisted multidrug coatings are still uncommon,^13,15^ even less in the VAP context. In this study, a novel coating strategy relying on the pDA-assisted co-immobilization of two antimicrobials, CHX and CIP, was exploited onto PVC surfaces. The definite mechanism for pDA synthesis is not well understood but it is thought to engage in two pathways, involving (i) covalent oxidation of catechol dopamine, which, in alkaline aqueous immersion, undergoes a balance with its quinone (amine) content, ultimately forming the dark brown insoluble biopolymer (pDA); (ii) noncovalent (physical) self-assembly of subproducts (products resulting from the covalent oxidative dopamine polymerization) forming H-bond and π–π interactions.^46^ Its ease of preparation has contrasted with the complex pDA formation process that varies widely on the reaction conditions^47^ and has generated diverse current theories of pDA structure and its formation.^48^ Nonetheless, the most well accepted and experientially evidenced is the coexistence of two active functional groups, catechol and amine,^12,28,48^ potentially contributing to the cross-linking of antimicrobials.

A deep characterization regarding surface properties (topography, roughness, wettability, molecular composition), the release profiles, and the potential for preventing microbial colonization while retaining the safety of use was made to unmodified vs modified PVC surfaces, so that we can go further with a great ETT coating near achieving success *in vivo*.

The extent of microbial adhesion and subsequent formation of biofilms are highly reliant on certain surface properties of polymeric substrates, such as wettability, roughness, and topography.^49^ Our prepared coatings imparted PVC surfaces with an altered homogeneously scattered morphology and varied wettability but a similar topography (low roughness) to pDA-coated surfaces. However, CIP/CHX coimmobilization yielded coatings with homogeneous, hydrophobic, and smooth topographic properties comparable to surfaces immobilized with CHX alone. This result is in accordance with Mohd Daud et al. that reported an increase in the hydrophobicity of stainless steel surfaces after CHX immobilization using a pDA-based approach.^35^ Although more research is needed, changes on coated surfaces compared to those on uncoated PVC relative to surface morphology and roughness may be related to antimicrobial aggregation^49^ and/or protrusions during polymerization of dopamine due to either water entrapment or complex polymer chain formation.^35^ ATR-FTIR spectroscopy corroborated the immobilization of CIP and/or CHX on PVC materials and still provided consistent information about the presence of hydroxyl and amine functional groups on the pDA structure, thus supporting the theory of pDA catechol and quinone forms coexisting.

In the design of antimicrobial coating strategies, the antimicrobial activity may be addressed either by a direct contact-killing approach, in which the antimicrobial compound is permanently attached to a surface, or by a strategy in which the antimicrobial agent is released.^50^ Although the first strategy has the advantage of decreasing the propensity for cytotoxicity and development of microbial resistance, there is a major setback associated with its application, namely, the fact that the first adherent dead cells may serve as a platform for the adhesion of the next ones, masking the antimicrobial effect of the surface. This is of particular concern if we bear in mind that the ETT biofilms, which often lead to severe VAP, are comprised of many different phylogenetic organisms and develop quickly after intubation.^41^ We, therefore, hypothesize that a dual-drug release appears to hold promise, in that it would benefit from a multitude of features, such as a long-lasting and broad-spectrum activity, thereby declining the emergence of antimicrobial resistance, reduction in antimicrobial doses, and good biocompatibility.^51^ It is important to note that there is no effective way to determine a reliable estimate of the ETT usage in the clinic, which may vary depending on several factors, e.g., the patient’s underlying medical condition and the purpose for intubation. For instance, during surgeries requiring general anesthesia, the ETT is usually removed shortly after the procedure is completed, so the duration of the application is limited to a few hours. On the other hand, the usage of ETT in ICU patients is expected to be prolonged, ranging from a few hours to several days. ETT exchange should be, however, avoided in patients with extremely difficult airways.^52^ While microbial colonization of the ETT may occur as early as a few hours after its insertion,^53^ and the rapid occlusion of the ETT lumen due to accumulative secretions may require emergency intervention/exchange, there are studies collecting ETTs for culture that had been indwelling for 12 h to 26 days.^53^ Another factor to take into consideration is the guidelines recommended by tube manufacturers. For instance, CeraShield ETT, developed by N8Medical, is recommended for adult patients intubated for times equal to or higher than 24 h, not specifying the exact duration for the intubation. Our findings showed a weighted but sustained release of CIP and CHX when immobilized on PVC surfaces, with both compounds (alone and combined) retaining contact antimicrobial activity for a prolonged period (up to 10 days).

Unlike a variety of emergent ETT antimicrobial coatings that have even considered the stability property, our dual-drug coating drives potential application for longer than 24 h, mitigating device failure for longer periods and consequently reducing the real risk for recurrent ETT exchanges and the propensity for developing VAP.

Biocompatibility, as well as stability, is another feature often dismissed in reports addressing antimicrobial materials for ETTs.^11^ Despite the efforts that have been made to study both of these traits, data remain scant or controversial, thereby leading to inconclusive safety and clinical usefulness of most emergent ETT coatings. It is the case, for instance, of silver-based ETT coatings. Even though they have rapidly reached the market with promising antimicrobial effectiveness, there is increasing evidence of the toxicity and short stability attained by released silver ions, in addition to the health concerns regarding the promotion of resistance in clinical isolates.^54^ When designing a dual-drug coating, in particular, it is imperative that the release of compounds is assured in doses that are high enough to provide an antimicrobial effect but not so that it will be cytotoxic. CHX toxicity toward mammalian cells has been reported to be dose-dependent^55^ and high intake of CIP can cause damage to internal organs, bleeding of the digestive system, and neurological diseases and allergies.^56^ In this study, coatings incorporating only CIP or CHX did not exhibit toxicity toward lung epithelial cells, even for the highest doses tested (2 mg/mL). In turn, surfaces co-immobilizing CIP/CHX caused A549 toxicity in a dose-dependent manner, and biocompatibility was only afforded for coatings incorporating antimicrobials with doses up to 0.5 mg/mL. Based on these findings, further antibiofilm studies were performed for coatings displaying no toxic effects against lung epithelial cells.

A key but considerably neglected factor has been the ETT biofilm and its polymicrobial etiology. Most antimicrobial coating strategies reported to fight VAP are tested against microorganisms individually.^57,58^ To the best of our knowledge, a sole polyurethane-based hydrogel ETT coating incorporating a lead ceragenin (CSA-131) was found to address performance against polymicrobial, including bacterial–bacterial (*P. aeruginosa*/MRSA) and bacterial–fungal (*P. aeruginosa*/*Candida auris*) biofilms.^59^ In addition, this latest study reported less efficient antimicrobial performance against polymicrobial biofilms compared to that of single-species consortia. The present study represents a step forward toward a collective representation of VAP pathogens while devising a dual-drug coating strategy to endow the surface of ETTs, with the ability to resist not only the colonization of Gram-negative (*P. aeruginosa*,*K. pneumoniae*, and*A. baumannii*) and Gram-positive bacteria (*S. aureus*,*S. epidermidis*) but also fungi (*C. albicans*) in single-, dual-, and triple-species consortia, thus reflecting worst-case scenarios in the context of VAP. PVC coatings with CHX-immobilized alone proved to be not effective against Gram-negative VAP-relevant bacteria, namely,*P. aeruginosa* and*A. baumannii*. In previous studies, ETTs impregnating CHX, particularly combined with other antiseptic agents, had demonstrated potential in reducing colonization not only by MDR Gram-negative bacteria but also by Gram-positive and *Candida* spp.^17^ The use of another antimicrobial compound, CIP, proved auspicious in broadening the antimicrobial spectrum of our coating strategy. CIP affords an antibacterial effect by binding to bacterial enzymes, DNA gyrase, and topoisomerase IV, resulting in permanent double-stranded DNA and cell death.^60^ Co-immobilization of CIP and CHX (CIP/CHX) imparted PVC surfaces with the ability to effectively prevent microbial colonization and biofilm formation by all five bacterial species investigated (*P. aeruginosa*,*A. baumannii*,*K. pneumoniae*,*S. aureus*, and*S. epidermidis*). In addition to increasing the spectrum of action and reducing antimicrobial resistance and toxicity, a combination of antimicrobials comprises additional advantages that include the rejuvenation of old antibiotics or drug synergism.^51^ In fact, CIP/CHX-modified surfaces exhibited both synergic and additive effects against all species, with reduced concentrations of both compounds crucial to ensure no toxicity against lung epithelial cells.

The proposed coating strategy was, therefore, further evaluated against polymicrobial consortia, namely, involving two and three species. Promising results led to the conclusion that the CIP/CHX mixed coating strategy exhibited similar or even better antimicrobial effects (regarding cell adhesion and viability) against polymicrobial communities when compared to their single-species colonization. The microbiome of VAP has been reported to be mainly comprised of bacteria and only a small percentage of viruses and fungi.^61^ The role played by such lesser common species, namely, fungal species such as*C. albicans*, on the adverse clinical outcomes in patients with VAP has not been fully elucidated. First, it was assumed that the presence of *Candida* spp. in respiratory tract specimens should be considered colonization rather than infection.^62^ In fact, ESCMID guidelines for the management of *Candida* spp. do not recommend antifungal therapy unless there is clear histological evidence of infection.^63^ However, other studies have suggested an association between *Candida* spp. colonization and longer mechanical ventilation,^64^ increased risk for multidrug-resistant bacterial isolation,^65^ and death.^66^ In the present study,*C. albicans* colonization as a single species was not impaired as efficiently as the biofilms of bacterial species. However, its presence did not compromise the antibiofilm activity against two major VAP pathogens,*P. aeruginosa* or*S. aureus*, when the coating strategies were evaluated against polymicrobial consortia.

Currently, the emergence of resistance to antibiotics and other antimicrobial agents is one of the greatest threats to human health. Despite the well-established antimicrobial efficiency of CIP and CHX, there has been a significant increase in reported resistance to both compounds.^67,68^ Consequently, another key factor in the development of antimicrobial release systems such as the one presented in this study is the potential development of resistance as a result of exposure to subinhibitory concentrations. Our dual-drug release system displayed a rapid initial release, effectively preventing the attachment of most species. In such strategies, the development of resistance should not be an issue, but further investigations are necessary to confirm this scenario. Another major limitation concerns the inability to estimate the exact amount of each immobilized compound through available absorbance-based methodologies due to interference by the one-step dopamine polymerization. Studies employing other techniques are further required to accurately measure the immobilization efficiency.

## 5 Conclusions

In this study, we followed a safe-by-design approach while integrating a multidisciplinary development process to design and implement a new antimicrobial material for improving ETT surfaces. While employing diverse practices and methodologies, we could obtain information on the physicochemical properties (morphology, wettability, roughness, molecular composition) of our coated surfaces and predict the successful pDA-assisted antimicrobial immobilization. Intriguingly, we could also attain a balance between antimicrobial features (at lowered doses) and biocompatibility for our novel dual-drug coating. The combination of CIP and CHX, using dopamine chemistry, resulted in a dual-drug coating endowed with broad-spectrum antimicrobial activity and with the capability of preventing microbial adhesion and affecting the cell integrity in more complex (interkingdom) polymicrobial scenarios without compromising the viability of lung epithelial cells. A sustained antimicrobial release for a period of up to 10 days evidenced the prolonged activity and robustness of the antimicrobial immobilization in our dual-drug coating.

Taking all findings together, our dual-drug coating approach holds great potential to be further applied in the next generation of EETs, with the promise of reducing the incidence rates of VAP.

## Supporting Information Available

Antimicrobial activity and qualitative release of CHX or CIP from PVC-modified surfaces; (A) microbial growth after 24 h contact with CHX- or CIP-modified surfaces, where + is indicative of the visible bacterial growth and – means no visible growth observed; (B) release of CHX or CIP on solid agar, qualitatively evaluated by the presence (P) or absence (A) of an inhibition zone; pDA-coated surfaces were also tested and used for comparison (Table S1); values of minimum inhibitory concentration (MIC) and minimum microbiocidal concentration (MMC) for chlorhexidine (CHX) and ciprofloxacin (CIP), expressed in mg/L, against planktonic cultures of *P. aeruginosa*, *A. baumannii*, *K. pneumoniae*, *S. aureus*, *S. epidermidis*, and *C. albicans*; the antimicrobial susceptibility of planktonic cultures was determined through the broth microdilution method, following the standard European Committee on Antimicrobial Susceptibility Testing (EUCAST) guidelines (Table S2); determination of the possible occurrence of facilitation or synergism for co-immobilization of CIP and CHX at a concentration of 0.5 mg/mL for single-species biofilm formation; the combinations where facilitation or synergism outcomes were obtained are highlighted in bold; in these equations, *C* refers to the microbial density obtained in the control (PVC surfaces) and *S*_CHX_, *S*_CIP_, and *S*_MIX_ refer to the surviving cell density after being in contact with surfaces functionalized with CHX, CIP, and the combination of CHX and CIP (MIX) (Table S3); determination of the possible occurrence of facilitation or synergism for co-immobilization of CIP and CHX at a concentration of 0.5 mg/mL for dual-species biofilm formation; the combinations where facilitation or synergism outcomes were obtained are highlighted in bold; in these equations, *C* refers to the microbial density obtained in the control (PVC surfaces) and *S*_CHX_, *S*_CIP_, and *S*_MIX_ refer to the surviving cell density after being in contact with surfaces functionalized with CHX, CIP, and the combination of CHX and CIP (MIX) (Table S4); surface roughness; AFM images of PVC surfaces before and after different pDA-based coating strategies for the immobilization of CIP and/or CHX: (A) unmodified PVC surface; (B) pDA coating; (C) CHX-modified surface with CHX at 0.5 mg/mL; (D) CHX-modified surface with CHX at 2 mg/mL; (E) CIP-modified surface with CIP at 0.5 mg/mL; (F) CIP-modified surface with CIP at 0.5 mg/mL; (G) CIP/CHX-modified surface with CIP and CHX at 0.5 mg/mL; (H) CIP/CHX-modified surface with CIP and CHX at 2 mg/mL (Figure S1); and effect of different selective media on bacterial and fungal growth; a microbial suspension of each microorganism investigated was adjusted to 1 × 10^6^ CFU/mL and plated on the different selective media (Figure S2) (PDF)
