# Correction to “Co-immobilization of Ciprofloxacin and Chlorhexidine as a Broad-Spectrum Antimicrobial Dual-Drug Coating for Poly(vinyl chloride) (PVC)-Based Endotracheal Tubes”

**DOI:** 10.1021/acsami.4c09227

**Published:** 2024-06-19

**Authors:** Diana Filipa Alves, Maria Olívia Pereira, Susana Patrícia Lopes

In the original version of the paper, Figure 4 lacked part of its legend and the respective data sets on the graphs. Specifically, the legend for CIP 0.5 mg/mL and its corresponding data set were omitted in Figure 4A, and the legend for CHX 0.5 mg/mL and its corresponding data set were also omitted in Figure 4B. With this correction, the profiles for the cumulative released masses of CIP and CHX, immobilized at different concentrations (0.5 mg/mL and 2 mg/mL), are now correctly illustrated in Figure 4A and B, respectively. This addition does not affect the conclusions or the discussion of the results presented in this work.

**Figure 4:**
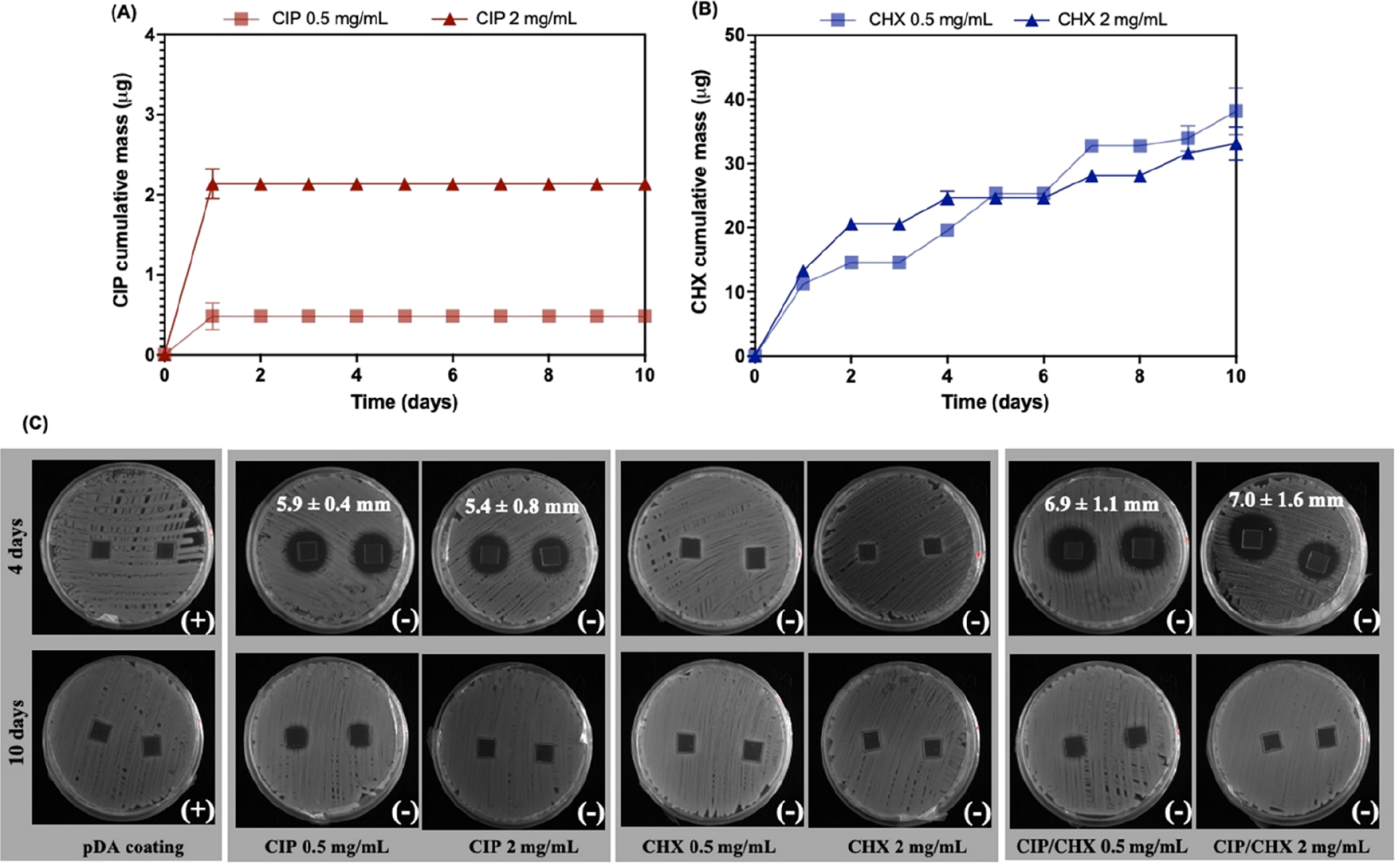
Antimicrobials release from coated surfaces. (A) Cumulative release of CIP and (B) of CHX from PVC surfaces functionalized with each compound at 0.5 and 2 mg/mL. Panel (C) depicts the outcomes from the contact antimicrobial activity and release of CIP or CHX immobilized alone, and CIP/CHX co-immobilized on PVC surfaces at 0.5 and 2 mg/mL, at days 4 and 10 against *K. pneumoniae*. Bacterial growth after 24 h contact with modified surfaces is indicated at the bottom right of each image, where “+” is indicative of visible bacterial growth and “–” means no visible growth observed. The release of antimicrobials on solid agar was evaluated by the presence or absence of an inhibition zone. The average length of inhibition zones was determined using ImageJ and presented in mm. pDA-coated surfaces were used for comparison.

The revised Figure 4, with the corrected graphics (Figures 4A and B), is provided below along with the respective caption.

In addition, the reference for the junior researcher contract of the author S. P. Lopes in the “Acknowledgements” section contains an error and should be considered as DL57/2016/CP1377/CT0043. Equally, this correction does not affect the conclusions or the discussion of the results presented in this work.

We regret this error and any confusion it may have caused. The original version of the article has been corrected to reflect these changes.

