# Supplementary material for: Co-immobilization of Ciprofloxacin and Chlorhexidine as a Broad-Spectrum Antimicrobial Dual-Drug Coating for Poly(vinyl chloride) (PVC)-Based Endotracheal Tubes
Source: ACS Appl Mater Interfaces. 2024 Mar 20;16(13):16861–79. doi: 10.1021/acsami.4c01334 (PMC10995906; doi:10.1021/acsami.4c01334)
Supplement: Supplementary file 1 — am4c01334_si_001.pdf [file am4c01334_si_001.pdf]

Supporting Information

Co-immobilization of ciprofloxacin and chlorhexidine as a broad-spectrum antimicrobial dual-drug coating for polyvinyl chloride (PVC)-based endotracheal tubes

Diana Alves[a,b,†], *Maria Olívia Pereira*[a,b], *Susana Patrícia Lopes*[a,b,*]

[a] CEB - Centre of Biological Engineering, University of Minho, 4710-057 Braga, Portugal

[b] LABBELS – Associate Laboratory, Braga/Guimarães, Portugal

†Present Address

†Diana Alves - INL - International Iberian Nanotechnology Laboratory, Av. Mestre José Veiga, 4715-330 Braga, Portugal

***Corresponding author**

*Susana P. Lopes: supat@ceb.uminho.pt

Table S1. Antimicrobial activity and qualitative release of CHX or CIP from PVC-modified surfaces. A) Microbial growth after 24 h contact with CHX- or CIP-modified surfaces, where "+" is indicative of visible bacterial growth and "–" means no visible growth observed. B) Release of CHX or CIP on solid agar, qualitatively evaluated by the presence (P) or absence (A) of an inhibition zone. pDA-coated surfaces were also tested and used for comparison

A)

		**CHX-modified PVC**		**CIP-modified PVC**	
	**pDA-coated surface**	**CHX 0.5 mg/mL**	**CHX 2 mg/mL**	**CIP 0.5 mg/mL**	**CIP 2 mg/mL**
P. aeruginosa	+	-	-	-	-
A. baumannii	+	+	-	+	-
K. pneumoniae	+	-	-	-	-
S. aureus	+	-	-	-	-
S. epidermidis	+	-	-	-	-
C. albicans	+	-	-	+	+

B)

		**CHX-modified PVC**		**CIP-modified PVC**	
	**pDA-coated surface**	**CHX 0.5 mg/mL**	**CHX 2 mg/mL**	**CIP 0.5 mg/mL**	**CIP 2 mg/mL**
P. aeruginosa	A	A	P	P	P
A. baumannii	A	A	A	A	P
K. pneumoniae	A	P	P	P	P
S. aureus	A	P	P	P	P
S. epidermidis	A	P	P	P	P
C. albicans	A	A	A	A	A

Table S2. Values of minimum inhibitory concentration (MIC) and minimum microbiocidal concentration (MMC) for chlorhexidine (CHX) and ciprofloxacin (CIP), expressed in mg/L, against planktonic cultures of *P. aeruginosa*, *A. baumannii*, *K. pneumoniae*, *S. aureus*, *S. epidermidis*, and *C. albicans*. The antimicrobial susceptibility of planktonic cultures was determined through the broth microdilution method, following the standard European Committee on Antimicrobial Susceptibility Testing (EUCAST) guidelines.

	**CHX**		**CIP**	
	**MIC**	**MMC**	**MIC**	**MMC**
P. aeruginosa	3.13 - 6.25	12.5 - 25	0.25	0.5 - 1
A. baumannii	12.5 - 25	25	2	4 - 8
K. pneumoniae	0.78 - 1.56	3.125	<0.016	<0.016
S. aureus	0.78 - 1.56	1.56 - 6.25	0.5	0.5 - 1
S. epidermidis	0.78 - 1.56	1.56 - 6.25	0.25	0.5 - 1
C. albicans	6.25 - 12.5	6.25 - 25	>64	>64

Table S3. Determination of possible occurrence of facilitation or synergism for co-immobilization of CIP and CHX at a concentration of 0.5 mg/mL for single-species biofilms formation. The combinations where facilitation or synergism outcomes were obtained are highlighted in bold. In these equations, C refers to the microbial density obtained in the control (PVC surfaces) and S_{CHX}, S_{CIP} and S_{MIX} to the surviving cell density after being in contact with surfaces functionalized with CHX, CIP, and the combination of CHX and CIP (MIX).

**Microorganism**	**Synergism**<br>[Log (S_C)– Log (S_{CHX}) – Log (S_{CIP}) + Log (S_{MIX})]	**Facilitation**<br>[Log (S_{MIX})– Log (S_{CHX}) / Log (S_{MIX}) - Log (S_{CIP})]
P. aeruginosa	**-4.908**	**-6.144 / -5.233**
A. baumannii	**-1.933**	**-4.161 / -1.885**
K. pneumoniae	5.393	**-0.167 / -0.083**
S. aureus	1.859	**-2.514 / -1.962**
S. epidermidis	6.534	**-0.620 / -0.033**
C. albicans	**-0.557**	**-0.283 / -0.753**

Table S4. Determination of possible occurrence of facilitation or synergism for co-immobilization of CIP and CHX at a concentration of 0.5 mg/mL for dual-species biofilms formation. The combinations where facilitation or synergism outcomes were obtained are highlighted in bold. In these equations, C refers to the microbial density obtained in the control (PVC surfaces) and S_{CHX}, S_{CIP} and S_{MIX} to the surviving cell density after being in contact with surfaces functionalized with CHX, CIP and the combination of CHX and CIP (MIX).

**Dual-species consortia**	**Synergism** [Log (S_C)– Log (S_{CHX}) – Log (S_{CIP}) + Log (S_{MIX})]	**Facilitation** [Log (S_{MIX})– Log (S_{CHX}) / Log (S_{MIX}) - Log (S_{CIP})]
P. aeruginosa	**-1.797**	**-6.449 / -2.040**
K. pneumoniae	4.732	**0 / 0**
P. aeruginosa	**-3.677**	**-7.349 / -4.138**
S. aureus	0.366	**-3.277 / -2.679**
P. aeruginosa	**-3.544**	**-6.791 / -3.797**
S. epidermidis	4.950	**-1.109 / 0.094**
P. aeruginosa	-2.909	**-7.267 / -2.579**
C. albicans	-0.853	**-1.853 / -1.711**

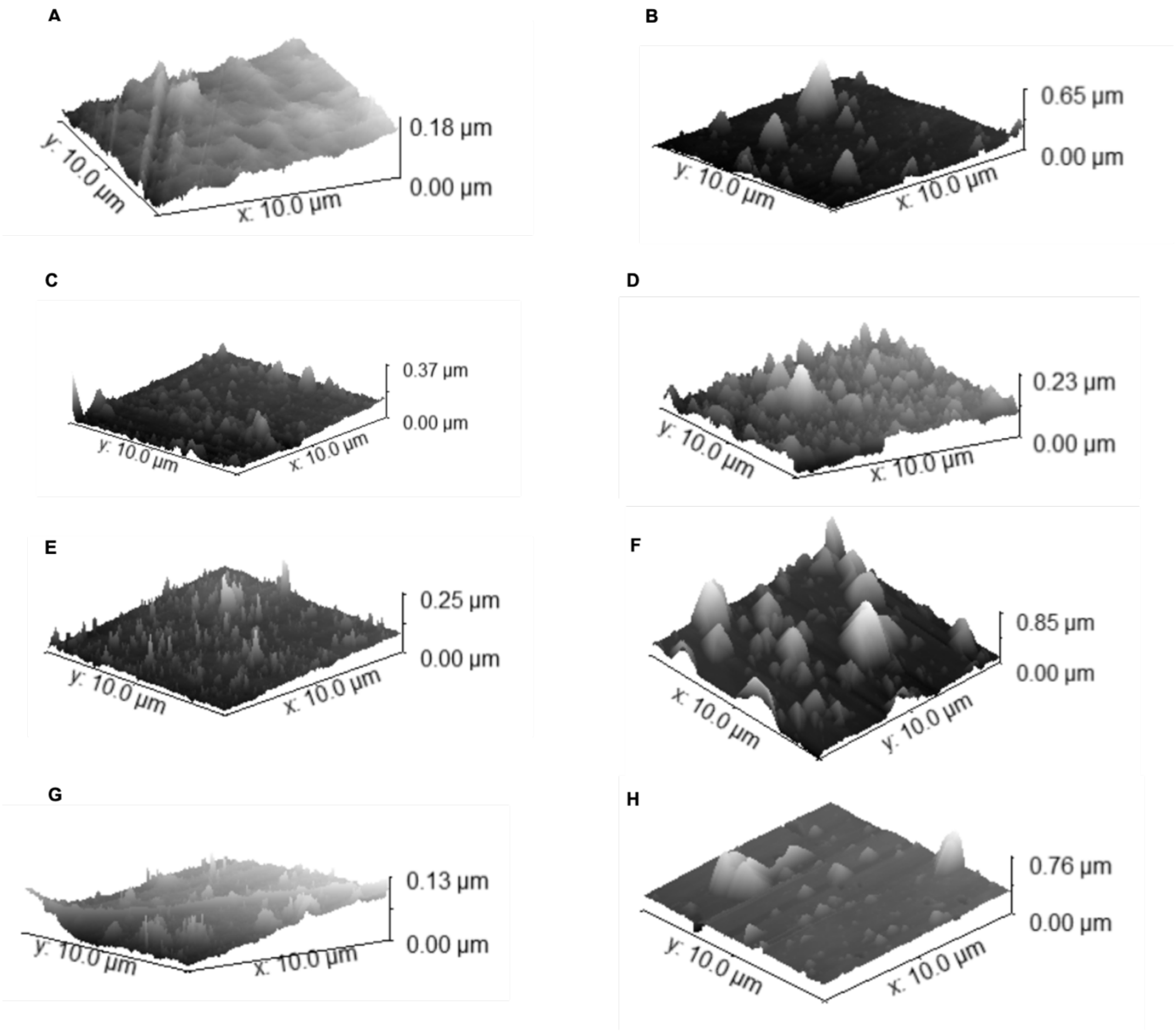

Figure S1. Surface roughness. AFM images of PVC surfaces before and after different pDA-based coating strategies for the immobilization of CIP and/or CHX: (A) unmodified PVC surface; (B) pDA coating; (C) CHX-modified surface with CHX at 0.5 mg/mL; (D) CHX-modified surface with CHX at 2 mg/mL; (E) CIP-modified surface with CIP at 0.5 mg/mL; (F) CIP-modified surface with CIP at 0.5 mg/mL; (G) CIP/CHX-modified surface with CIP and CHX at 0.5 mg/mL; (H) CIP/CHX-modified surface with CIP and CHX at 2 mg/mL.

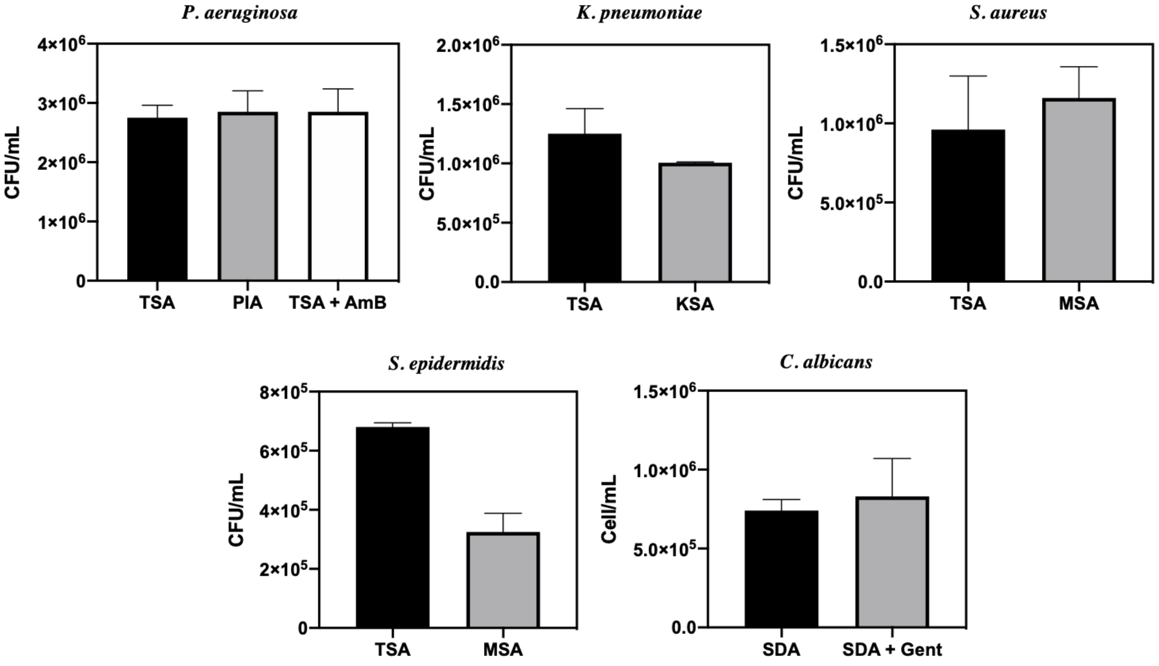

Figure S2. Effect of different selective media on bacterial and fungal growth. A microbial suspension of each microorganism investigated was adjusted to $1x10^6$ CFU/mL and plated on the different selective media.
